# Inhibition of ATM enhances the immunogenicity of triple-negative breast cancer by promoting MHC-I expression

**DOI:** 10.1038/s41419-025-07944-y

**Published:** 2025-08-18

**Authors:** Jiazhen Li, Chenying Liu, Xiaolong Qian, Xiaozi Wang, Hui Sun, Lu Wang, Huiqin Xue, Yuanming Song, Jiamei Liu, Yafang Zhao, Yumian Jia, Fengxia Qin, Tianhua Zhang, Xiaojing Guo

**Affiliations:** Department of Breast Pathology and Lab, Tianjin Medical University Cancer Institute & Hospital, National Clinical Research Center for Cancer, Key Laboratory of Breast Cancer Prevention and Therapy of Ministry of Education of China, Tianjin Medical University, Tianjin’s Clinical Research Center for Cancer, West Huanhu Road, 300060, Tianjin, China

**Keywords:** Breast cancer, Tumour immunology, Cancer microenvironment

## Abstract

The immunotherapy has achieved some efficacy in triple-negative breast cancer (TNBC), but the benefit population is limited, primarily due to an abnormal immune microenvironment. Thus, it is necessary to explore new molecular targets to enhance the immunogenicity of TNBC cells and improve their responsiveness to immunotherapy. We found that a key component of the DNA repair system, Ataxia telangiectasia mutated (ATM), may function as an immune response inhibitor. In this study, the inverse correlation between ATM and CD8^+^ T cells and tumor-infiltrating lymphocytes (TILs) was confirmed by immunochemical staining of 191 TNBC specimens. Subsequently, inhibition of ATM increased the expression of major histocompatibility complex I (MHC-I) and enhanced the infiltration and cytotoxic activity of CD8^+^ T cells by Western blot and flow cytometry analysis. In addition, we further confirmed that the MHC-I upregulation induced by ATM inhibition depends on the activation of the c-Jun/TNF-α/p-STAT1 pathway. Animal studies have shown that ATM deficiency delays tumor growth and sensitizes tumors to PD-1 blockade and radiotherapy. This study reveals a new mechanism by which ATM negatively regulates MHC-I by inhibiting the c-Jun/TNF-α/p-STAT1 pathway in TNBC, and shows an important role in mediating CD8^+^ T cells infiltration and regulating the “heat” of the immune microenvironment. The combination of ATM inhibitors with radiotherapy and Immune-checkpoint blockade (ICB) therapies may be a new strategy for TNBC treatment.

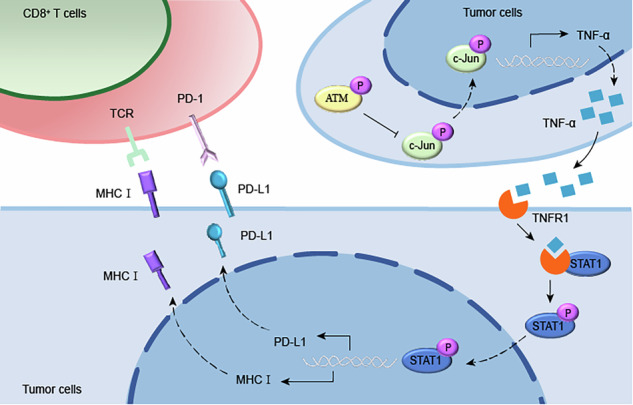

## Introduction

TNBC represents the most aggressive molecular subtype of breast cancer, constituting 10-20% of all cases, and is associated with a poor prognosis due to a lack of specific therapeutic targets [1]. While chemotherapy remains the primary treatment for TNBC, it is often hindered by drug resistance and off-target toxicity [2]. In recent years, immunotherapy has emerged as a promising treatment for TNBC. Multiple immune checkpoint inhibitors (ICIs) have been approved for the treatment of triple-negative breast cancer, leading to significant improvements in patient outcomes [3–5]. The PD-1/PD-L1 axis plays a crucial role in dampening T cell activity and facilitating tumor immune evasion [6]. Pembrolizumab monotherapy was confirmed to provide sustained antitumor activity in patients with early and advanced PD-L1-positive TNBC (combined positive score [CPS] ≥1) [7]. Additionally, a Phase I clinical trial (NCT02838823) highlighted the safety and efficacy of the PD-1 inhibitor JS001 in patients with metastatic TNBC who had previously undergone multiple treatment lines [8].

The inactive tumor microenvironment is the main reason for the poor efficacy of chemotherapy and immunotherapy [9]. Studies have shown that TILs are significantly associated with improved prognosis in TNBC patients [10, 11]. When the expression level of MHC-I—known as human leukocyte antigen (HLA) in humans and histocompatibility system 2 (H-2) in mice—within tumor cells is down-regulated or absent, the cytotoxic effect mediated by CD8^+^ T cells can be affected, allowing tumor cells to evade immune surveillance and clearance [12, 13]. Numerous studies have shown that PD-1/PD-L1 inhibitor treatment is more effective in cancers characterized by a higher presence of CD8^+^ T cells, which can serve as predictive and therapeutic biomarkers for anti-PD-1 therapy [14, 15]. Therefore, enhancing the recruitment of CD8^+^ T cells and remodeling the immune microenvironment are important methods to improve the therapeutic effect of ICIs in breast cancer.

The ATM gene encodes a serine/threonine protein kinase, which plays an important role in DNA damage response (DDR) and cell cycle regulation [16, 17]. ATM plays a key role in the detection and repair of DNA double-strand breaks (DSBS) caused by ionizing radiation (IR) [18, 19]. ATM-deficient cells are extremely sensitive to IR, so ATM inhibitors have been explored as sensitizers for radiotherapy [20]. Additionally, studies have found that ATM is highly expressed in ER-negative breast cancer, with its expression level inversely correlated with CD8^+^ T cells infiltration [21, 22]. This suggests that ATM deficiency or inhibition may enhance the antitumor effects of radiotherapy as well as ICB therapies [23, 24]. Defects in the DDR pathway may increase the immunogenicity of tumor cells and improve the effectiveness of ICIs [25–28]. Moreover, both alterations in the DDR pathway and radiotherapy have been shown to induce the expression of immune checkpoint ligands such as PD-L1, which provides an opportunity for the intervention of ICIs [27, 29, 30].

In this study, we performed single-cell sequencing and immunohistochemical (IHC) analysis of tumor tissues from TNBC patients and found that the expression of ATM was significantly negatively correlated with the expression of TILs, MHC-I and CD8^+^ T cells. Additionally, we found that inhibition of ATM can up-regulate the expression of MHC-I through c-Jun/TNF-α/p-STAT1, thereby enhancing CD8^+^ T cells infiltration and increasing the immune “heat” in TNBC. It further proves the effectiveness of the combination of ATM inhibitors with radiotherapy and ICB therapies, providing a new strategy for enhancing the efficacy of cancer treatment.

## Materials and methods

### Human peripheral blood mononuclear cell (PBMC) isolation and co-culture with tumor cells

Peripheral venous blood (10 mL) was collected from fasting human subjects using EDTA-anticoagulated vacuum tubes. Within 4 h of collection, the whole blood was diluted 1:1 with phosphate-buffered saline (PBS) and subjected to density gradient centrifugation using Ficoll-Paque solution (Solarbio, China) at 1200 × *g* for 15 min at room temperature for PBMC isolation. Collect PBMCs in a separate tube by carefully pipetting the cells from the layer. Cells were then washed with an equal volume of PBS and centrifuged at 1200 × *g* for 15 min. This washing procedure was repeated twice to obtain purified PBMCs.T cells were activated with plate-bound anti-human CD3 (2 µg/mL, clone OKT3) and anti-human CD28 (2 µg/mL, clone CD28.2) antibodies (BioLegend, San Diego, CA, USA) in the presence of 100IU/mL interleukin-2 (PROSPEC, East Brunswick NJ, USA). T cells were cultured in 25 cm^2^ cell culture flasks with RPMI 1640 medium (Gibco) in a total volume of 5 mL and maintained at 37 °C in 5% CO_2_ atmosphere. The cells were diluted up to 5 × 10^5^ cells/mL by adding fresh culture medium supplemented with 25IU/mL interleukin-2 on days 4, 7 and 10. Informed consent was obtained from all volunteers. Subsequently, the acquired PBMCs were co-cultured with tumor cells at a ratio of 10:1 for 48 h to assess immune alterations.

### Lactate dehydrogenase (LDH) cytotoxicity assay

Cytotoxicity of CD8^+^ T cells was determined by LDH Cytotoxicity Assay Kit (Beyotime Biotechnology). TNBC-shV and TNBC-shATM cells were cultured normally and diluted into 2 × 10^4^/100 μL using 1640 complete medium after trypsin digestion. At the same time, the PBMC cells sorted from the above experimental procedure were resuspended in 1640 complete medium, and four gradients were set up, and the concentration was 1 × 10^5^/100 μL (target cell: effector cell = 1:5), 2 × 10^5^/100 μL (target cell: effector cell = 1:10), 3 × 10^5^/100 μL (target cell: effector cell = 1:15) and 4 × 10^5^/100 μL (target cell: effector cell = 1:20). Prepare the 96-well cell culture plate and design the following groups with 3 repeat holes in each group:

- Background control: 200 μL medium.
- Low control: 100 μL tumor cells+100 μL medium.
- High control: 100 μL tumor cells+100 μL Membrane breaking solution (2% Triton-X100 in culture medium).
- Effector control: 100 μL CD8^+^ T cells+100 μL medium.
- Experimental group (Effector/target cell mix) : 100 μL tumor cells +100 μL CD8^+^ T cells (different proportions).

After spreading the plates for a total of 20 h, the culture solution was transferred to a 96-well plate and mixed with the reaction mixture for 30 min at room temperature. Following the addition of stop solution, the OD490 and OD680 values were assayed, and the difference (OD490-OD680) represented LDH activity. Cytotoxicity (%) = (Effector/target cell mix -Effector cell control–Low control)/(High control-Low control) × 100.

### Flow cytometry

Cells were harvested and collected by centrifuging at 1,500 rpm for 5 min. For molecular staining of cell surfaces, resuspended cells were directly incubated with the related antibody and protected from lights on ice for 30 min and then washed with staining buffer. For intracellular molecular staining, the BD cell fixation and permeabilization were used for the fixation of cells and the Perm/Wash Buffer was used to wash the cells and to dilute the antibodies for staining (BD, no. 554714). The supernatant was aspirated, and the cells were resuspended in 200 μL staining buffer for flow cytometric analysis. The following antibodies were used for flow cytometry analysis. Brilliant Violet 510™ anti-human CD45 Antibody (biolegend, no. 304035), APC/Fire™ 750 anti-human CD3 Antibody(biolegend, no. 300469), Brilliant Violet 785™ anti-human CD8 Antibody(biolegend, no. 344739), PE anti-human CD279 (PD-1) Antibody(biolegend, no. 329905), Brilliant Violet 421™ anti-human CD366 (TIM-3) Antibody(biolegend, no. 345007), FITC anti-human IFN-γ Antibody(biolegend, no. 502505), BUV395 anti-human TNF(BD Biosciences, no. 563996), Anti-MHC class I + HLA A + HLA B antibody(abcam, ab134189), All the flow cytometry data were processed with FlowJo software (version 10.9).

### Immunohistochemical procedure

IHC analysis on formalin-fixed tissue sections was performed with an avidin-biotin system using a standard protocol. The antibodies used are as follows: ATM rabbit monoclonal antibody (mAb) (Abcam, ab32420), 1:100 dilution; MHC class I + HLA A + HLA B rabbit mAb (Abcam, ab134189), 1:4000 dilution; CD8-α mouse mAb (Santa Cruz Biotechnology, sc-7970), 1:100 dilution; TNF-α mouse mAb (proteintech, 26405-1-AP), 1:200 dilution; Phospho-Stat1 rabbit mAb (Tyr701) (Cell Signaling Technology, 9167 T), 1:400 dilution. Phospho-ATM S1981 recombinant rabbit mAb(HuaBio, ET1705-50), 1:200 dilution; HLA-A + HLA-B + HLA-C rabbit pAb(Abclonal, A1285) 1:100 dilution. The sections were then incubated sequentially with primary antibody, biotinylated secondary antibody, and avidinperoxidase conjugate. All steps were preceded by rinsing sections with PBS (pH 7.6). The chromogen was DAB.

### Clinical specimens and ethical approval

The 191 samples used in this study were all surgical resection specimens of TNBC admitted to Tianjin Medical University from August 2014 to December 2016 without preoperative neoadjuvant chemotherapy, and confirmed to be pathological stage II and III after surgery. Detailed clinicopathological information is shown in Table S3. All human breast tissues were collected with written consent from patients prior to participation in the study. All studies involved in this study meet the requirements of the Ethics Committee.

### Additional materials and methods

Additional methods are described in Supplementary Materials and Methods.

## Results

### The expression of ATM is negatively correlated with TILs and CD8^+^ T cells in TNBC

**Fig. 1:**
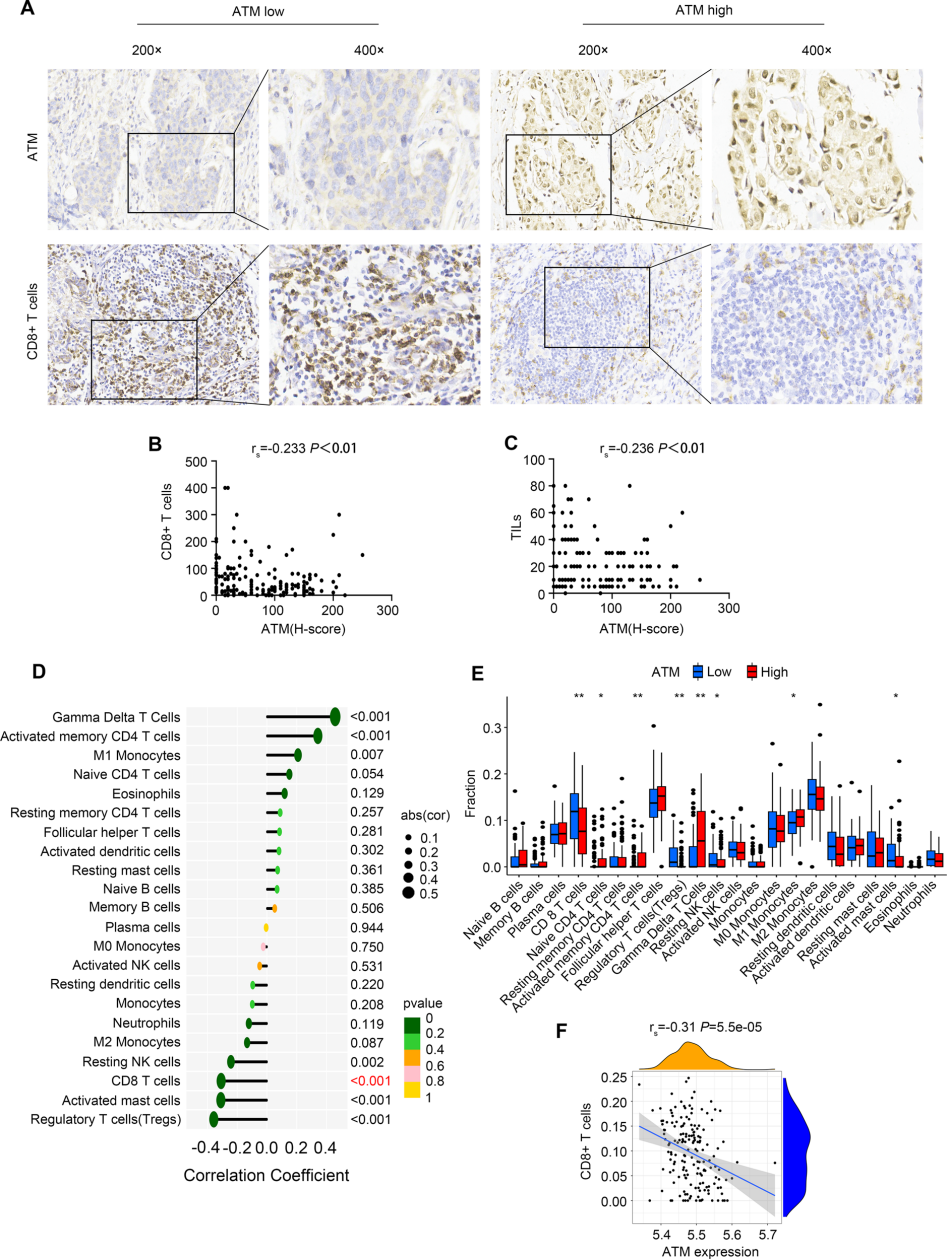
The expression of ATM is negatively correlated with TILs and CD8^+^ T cells in TNBC. **A** TNBC tissues were stained with ATM and CD8^+^ T cells, representative IHC figures were shown (200 ×). **B** Correlation of ATM expression with CD8^+^ T cell expression. **C** Correlation of ATM expression with TILs expression. **D**, **E** Associations between ATM expression and immune cells were analyzed in publicly available datasets (GSE76124). **F** Association between ATM expression and CD8^+^ T cell in publicly available datasets (GSE76124).

We performed immunohistochemical staining on samples from 191 TNBC patients (Fig. 1A). Quantitative evaluation demonstrated that ATM expression levels did not show statistically significant differences when compared across pathological stages (stage II vs stage III, *P* = 0.674), histological grades (grade 1/2 vs grade 3, *P* = 0.972), or between special and non-special histological subtypes (*P* = 0.063).

The raw scores from the IHC analysis showed that the expression of ATM was negatively correlated with the expression of CD8^+^ T cells (*r*_s_ = −0.233, *P* = 0.001) and TILs(*r*_s_ = −0.236, *P* = 0.001) (Fig. 1B, C). This negative correlation was consistent in subgroups of patients with different pathological stages (105 cases in stage II, 86 cases in stage III) and histological grades (66 cases in grade 1/2, 113 cases in grade 3) (Table S4 and S6).

To investigate the relationship between ATM expression levels and immune cells in the tumor microenvironment, we analyzed data from 198 TNBC samples in GEO data (GSE76124). We found a significant negative correlation between ATM and CD8^+^ T cells, supporting our previous IHC findings (Fig. 1D–F). These results suggest that ATM may serve as a potential regulatory molecule influencing the immune “heat” of TNBC.

### ATM knockdown is expected to improve the immune “heat” of TNBC

**Fig. 2:**
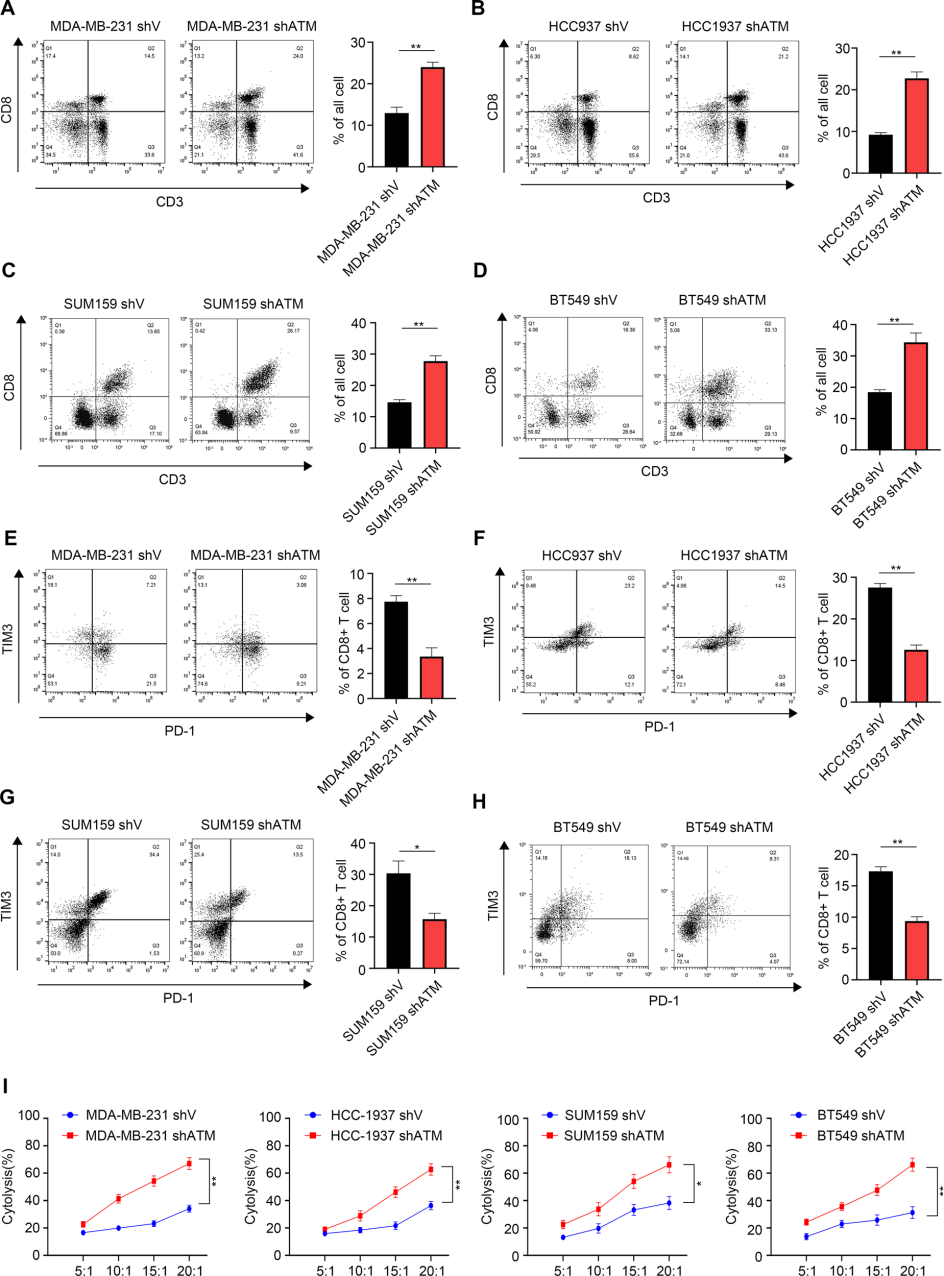
ATM knockdown is expected to improve the immune “heat” of TNBC. **A**–**H** Representative dot plots (left) and statistical analysis (right) of CD8^+^ T cells (**A**–**D**), CD8^+^ T cells exhaustion (**E**–**H**) cocultured with the indicated cell line. *P < 0.05 and **P < 0.01, by unpaired *t* test. The experiments were performed and repeated at least three times, independently. **I** The killing efficiency of different proportions of CD8^+^ T cells to tumor cells was detected by LDH killing kit. Error bars represent mean ± SD. *P < 0.05 and **P < 0.01, two-way analysis of variance (ANOVA).

To clarify the effect of ATM knockdown on CD8^+^ T cells, PBMC extracted from the peripheral blood of healthy volunteers was co-cultured with tumor cells in vitro. After 24 h of co-culture, the changes of immune cells in the supernatant were detected by flow cytometry. Notably, we observed a significant upregulation in the proportion of CD3^+^ CD8^+^ T cells following ATM knockdown in TNBC cell lines MDA-MB-231, HCC1937, SUM159 and BT549 (Fig. 2A–D). In addition, the percentage of IFN-γ^+^ CD8^+^ T cells and TNF-α^+^ CD8^+^ T cells were both increased in the TNBC-shATM cells compared with the control TNBC-shV cells (Fig. S1A–D). At the same time, the percentage of PD-1^+^ TIM-3^+^ exhausted CD8^+^ T cells was reduced in TNBC-shATM cells (Fig. 2E–H).

Moreover, we observed the recognition and killing of tumor cells by CD8^+^ T cells at different time points of co-culture. At the 20-hour mark, TNBC-shATM cells were recognized and killed by more CD8^+^ T cells than TNBC-shV cells. Subsequently, we used the LDH cytotoxicity assay to detect the killing efficiency of CD8^+^ T cells against tumor cells at the 20th hour of co-culture under different gradients. Compared to TNBC-shV cells, the killing efficiency of CD8^+^ T cells against TNBC-shATM cells was significantly increased and improved with the rising proportion of CD8^+^ T cells (Fig. 2I). These results indicate that ATM knockdown can enhance the immune function of CD8^+^ T cells and their killing ability against tumor cells.

### ATM deficiency in TNBC cells promotes MHC-I expression

**Fig. 3:**
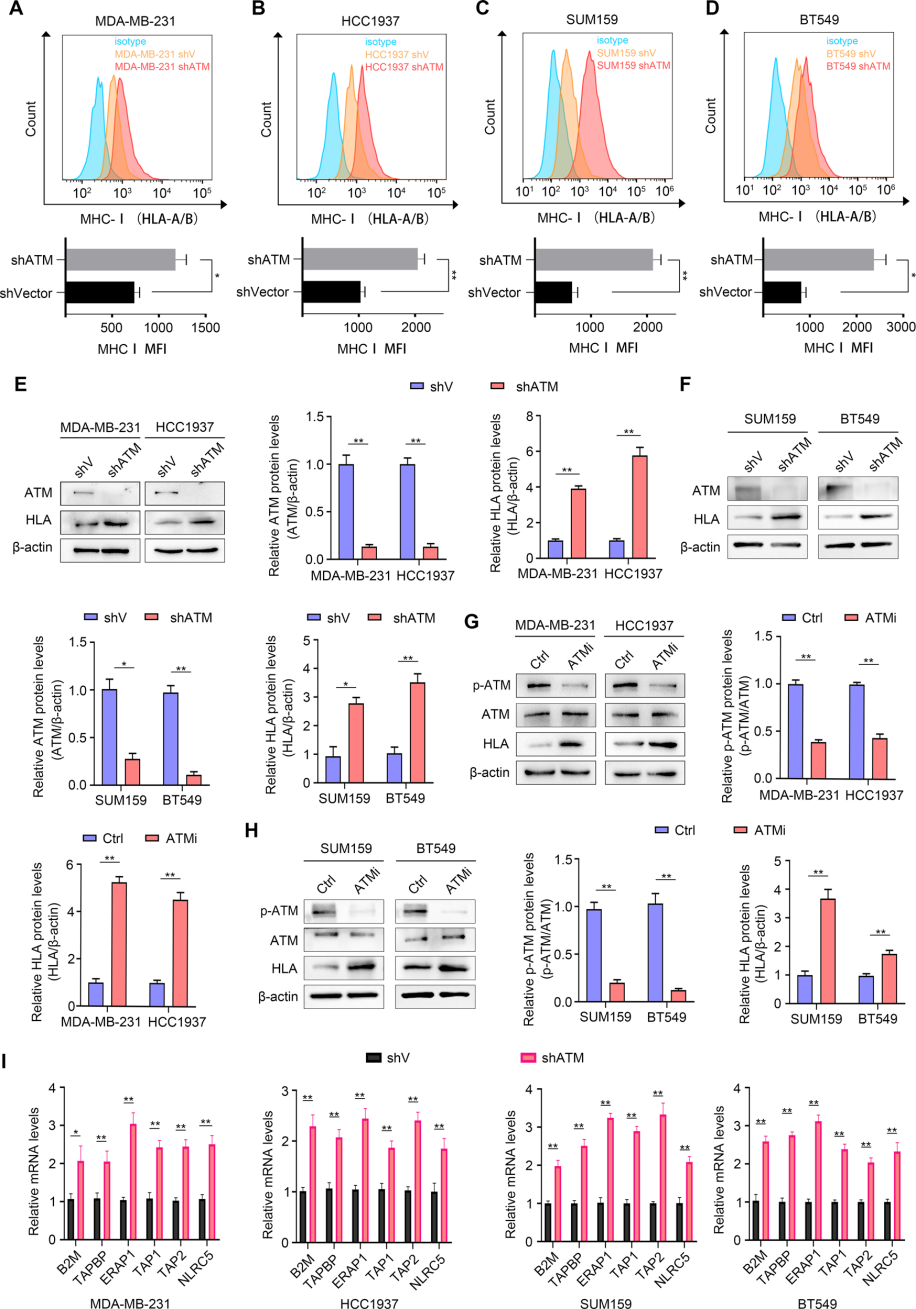
ATM deficiency in TNBC cells promotes MHC-I expression. **A**–**D** Representative histogram (top) and statistical analysis (bottom) of HLA expression. *P < 0.05 and **P < 0.01, by unpaired *t* test (**E**, **F**). Western blot analyses showed the effect of silencing ATM on the protein expression of ATM and HLA in MDA-MB-231, HCC1937, SUM159 and BT549 cells with indicated treatments. Quantitative of relative ATM and HLA protein levels. **G**, **H** Western blot analyses showed the effect of ATM inhibition on the protein expression of p-ATM (S1981), ATM, and HLA in MDA-MB-231, HCC1937, SUM159 and BT549 cells with indicated treatments. Quantitative of relative p-ATM (S1981) and HLA protein levels. ATMi (ATM inhibitor KU55933, 10 μM). **I** qRT-PCR analyses showed the effects of ATM knockdown on B2M, TAPBP, ERAP1, TAP1, TAP2 and NLRC5 mRNA levels in MDA-MB-231 and HCC1937 cells. Error bars represent mean ± SD. Two-tailed Student’s *t* test was used for statistical analysis. *P < 0.05; **P < 0.01; ns, not significant, *P* > 0.05.

To further dissect the mechanism of the negative correlation between ATM and CD8^+^ T cells, we noted that MHC-I, as a key molecule of antigen presentation, plays an indispensable role in the activation process of CD8^+^ T cells. We speculate that ATM may affect the recognition and response of CD8^+^ T cells to specific antigens by regulating the expression level of MHC-I (known as HLA in humans) molecules or the efficiency of antigen presentation. To test this hypothesis, we first constructed ATM knockdown stable cell lines in four TNBC cell lines, MDA-MB-231, HCC1937, SUM159 and BT549, and found that the expression of MHC-I (HLA-A + HLA-B) on the surface of tumor cells was increased by flow cytometry (Fig. 3A–D). In addition, we observed that both the protein and mRNA levels of HLA were upregulated after ATM knockdown (Figs. 3E, F, and S2A, B). Similarly, treatment of TNBC cells with KU55933 also increased the protein expression and mRNA levels of HLA (Figs. 3G, H, and S2C, D). These findings suggest that ATM negatively regulates the transcriptional activation and protein expression of MHC-I in TNBC cells in vitro.

We also confirmed that in TNBC-shATM, Genes encoding key components of MHC-I (β2m), MHC-I loading machinery (Erap1, Tap 1, and Tap2), and MHC-I transcriptional coactivator (NLRC5) were upregulated at the transcriptional level relative to TNBC-shV (Fig. 3I). These results suggest that ATM can negatively regulate HLA expression in TNBC.

### ATM knockdown upregulates MHC-I expression through TNF-α in TNBC

**Fig. 4:**
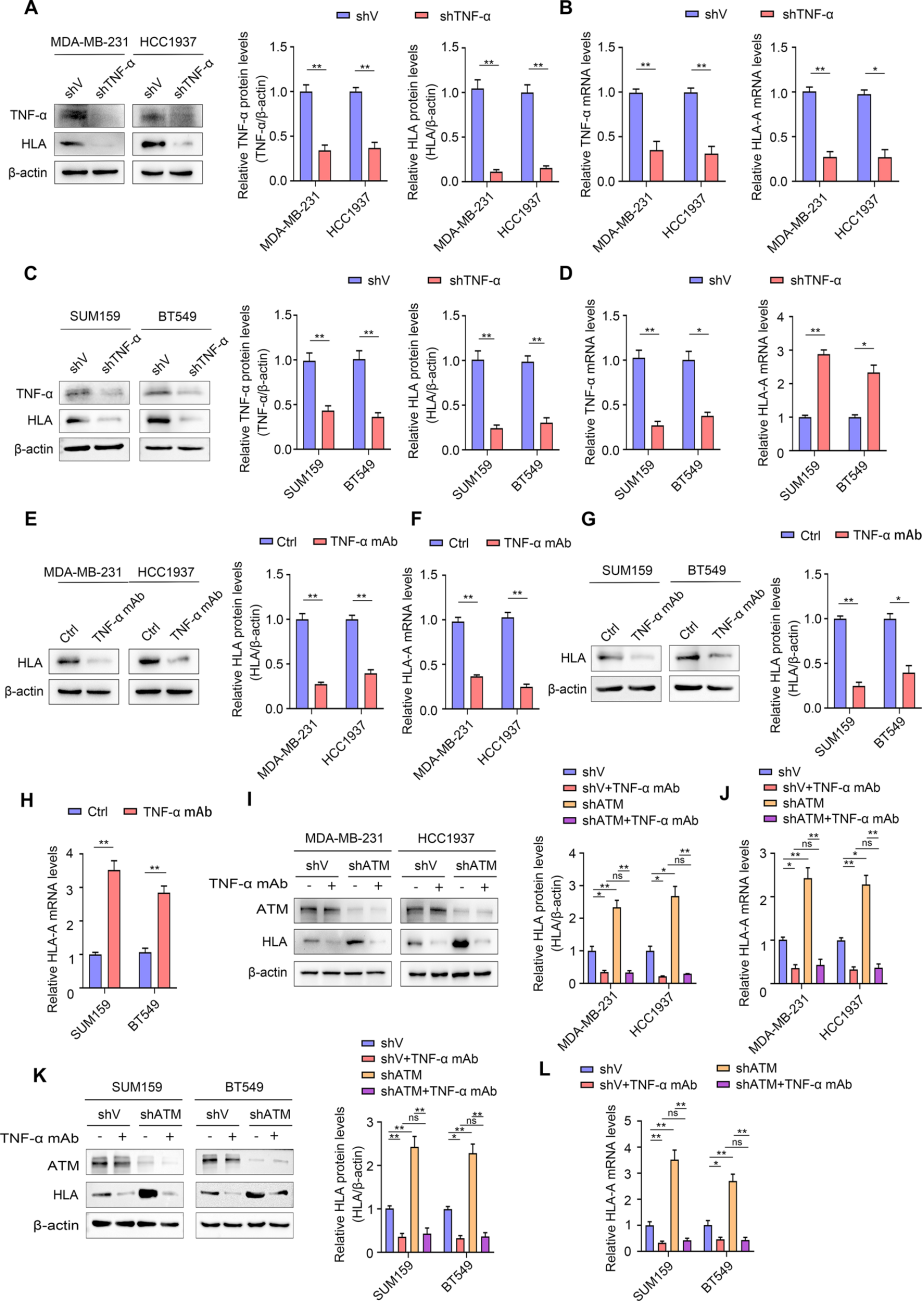
ATM knockdown upregulates MHC-I expression through TNF-α in TNBC. **A**, **C** Western blot analyses showed the effect of silencing TNF-α on the protein expression of TNF-α and HLA in MDA-MB-231, HCC1937, SUM159 and BT549 cells with indicated treatments. Quantitative of relative TNF-α and HLA protein levels. **B**, **D** qRT-PCR analyses showed the effect of silencing TNF-α on the mRNA levels of TNF-α and HLA-A in MDA-MB-231, HCC1937, SUM159 and BT549 cells with indicated treatments. **E**, **G** Western blot analyses showed the effect of TNF-α monoclonal antibody on the expression of HLA protein in MDA-MB-231, HCC1937, SUM159 and BT549 cells. Quantitative of relative HLA protein levels. **F**, **H** qRT-PCR analyses showed the effect of TNF-α monoclonal antibody on HLA-A mRNA levels in MDA-MB-231, HCC1937, SUM159 and BT549 cells. **I**, **K** Protein expression by Western blot analyses of ATM and HLA in MDA-MB-231, HCC1937, SUM159 and BT549 cells with indicated treatments. Quantitative of relative HLA protein levels. **J**, **L** mRNA levels by qRT-PCR analyses of HLA-A in MDA-MB-231, HCC1937, SUM159 and BT549 cells with indicated treatments. Error bars represent mean ± SD. Two-tailed Student’s *t* test was used for statistical analysis. *P < 0.05; **P < 0.01; ns, not significant, *P* > 0.05.

Subsequently, we sought to investigate the key mechanism underlying the regulation of MHC-I expression by ATM. Some studies have shown that TNF-α can up-regulate the expression of MHC-I by activating the NF-κB signaling pathway [31, 32]. Previous studies have confirmed that ATM knockdown induces up-regulation of TNF-α expression [33]. Therefore, we hypothesized that ATM knockdown mediates elevated MHC-I expression through TNF-α. To test this hypothesis, Western blot and qRT-PCR analyses were performed to assess changes in HLA expression in the TNBC-shV and TNBC-shTNF-α cell lines. The results showed that after the knockdown of TNF-α in TNBC cells, the protein expression and mRNA levels of HLA were down-regulated (Fig. 4A–D). Consistent conclusions were obtained when we treated TNBC cells with TNF-α mAb (Fig. 4E–H). In TNBC-shATM cells, the knockdown of ATM increased the expression of HLA compared with TNBC-shV cells, however, when TNF-α was blocked, the upregulated expression of HLA were significantly inhibited (Fig. 4I, K). The changes in the mRNA levels of HLA-A are consistent with the protein expression (Fig. 4J, L).

### C-Jun-mediated TNF-α/p-STAT1 activation induces MHC-I expression in ATM knockdown TNBC

**Fig. 5:**
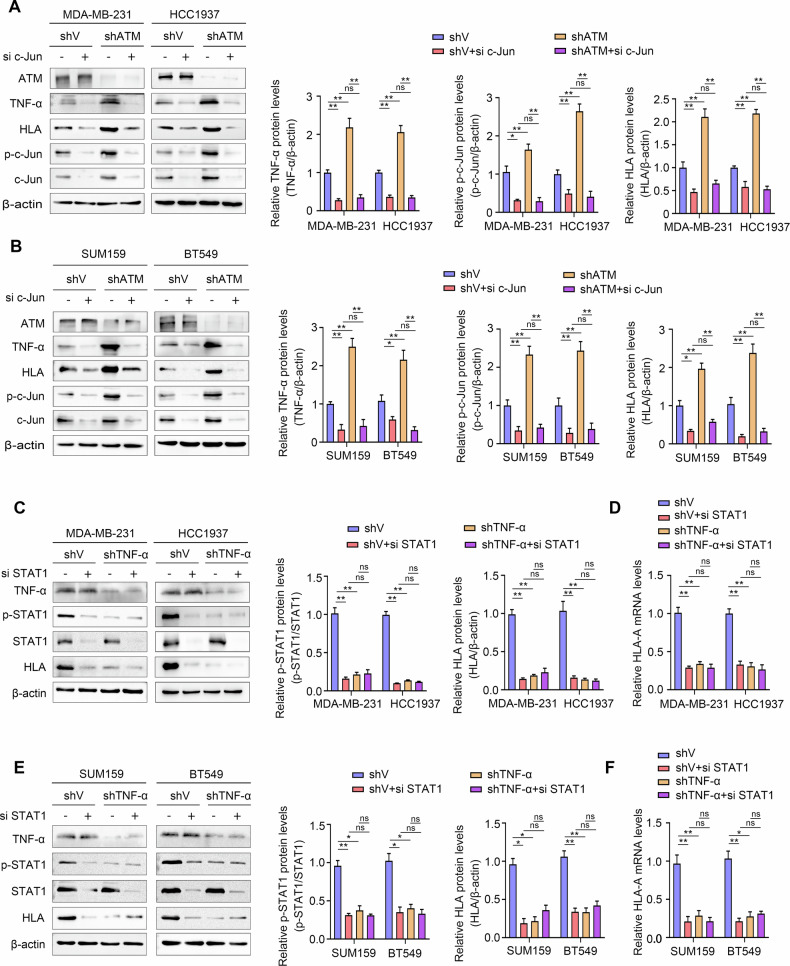
C-Jun-mediated TNF-α/p-STAT1 activation induces MHC-I expression in ATM knockdown TNBC. **A**, **B** Protein expression by Western blot analyses of ATM, TNF-α, HLA, p-c-Jun and c-Jun in MDA-MB-231, HCC1937, SUM159 and BT549 cells with indicated treatments. Quantitative of relative TNF-α, p-c-Jun and HLA protein levels. **C**, **E**) Protein expression by Western blot analyses of TNF-α, p-STAT1, STAT1 and HLA in MDA-MB-231, HCC1937, SUM159 and BT549 cells with indicated treatments. Quantitative of relative p-STAT1 and HLA protein levels. **D**, **F** mRNA levels by qRT-PCR analyses of HLA-A in MDA-MB-231, HCC1937, SUM159 and BT549 cells with indicated treatments. Error bars represent mean ± SD. Two-tailed Student’s *t* test was used for statistical analysis. *P < 0.05; **P < 0.01; ns, not significant, *P* > 0.05.

Previous studies have demonstrated that ATM deficiency promotes c-Jun phosphorylation, leading to its nuclear translocation and subsequent binding to the TNF-α promoter region, thereby enhancing TNF-α transcription and expression [33]. Therefore, we further verified whether c-jun affects the expression of MHC-I through TNF-α. In TNBC-shATM cells, the knockdown of ATM increased the expression of p-c-Jun, TNF-α, and HLA compared with TNBC-shV cells, however, when c-Jun was silenced, the upregulated expression of both TNF-α and HLA was significantly suppressed (Fig. 5A, B). This suggests that c-Jun plays an important role in this regulatory network.

Prior work has shown that TNFR1-STAT1 interaction and p-STAT1 expression are significantly increased upon TNF-α stimulation [34]. The activation of STAT1 can promote the up-regulation of MHC-I [35, 36]. We used siRNA to silence STAT1 and confirmed its impact on HLA expression in TNBC-shV cells. In TNBC-shTNF-α cells, knockdown of TNF-α reduced the expression of HLA and p-STAT1 compared with TNBC-shV cells. Silencing STAT1 further reduced the protein expression and mRNA levels of HLA (Fig. 5C–F). In conclusion, ATM knockdown upregulates HLA expression in triple-negative breast cancer through activation of the c-Jun/TNF-α/p-STAT1 signaling pathway.

### The expression of ATM was negatively correlated with TNF-α, p-STAT1 and MHC-I in TNBC patients

**Fig. 6:**
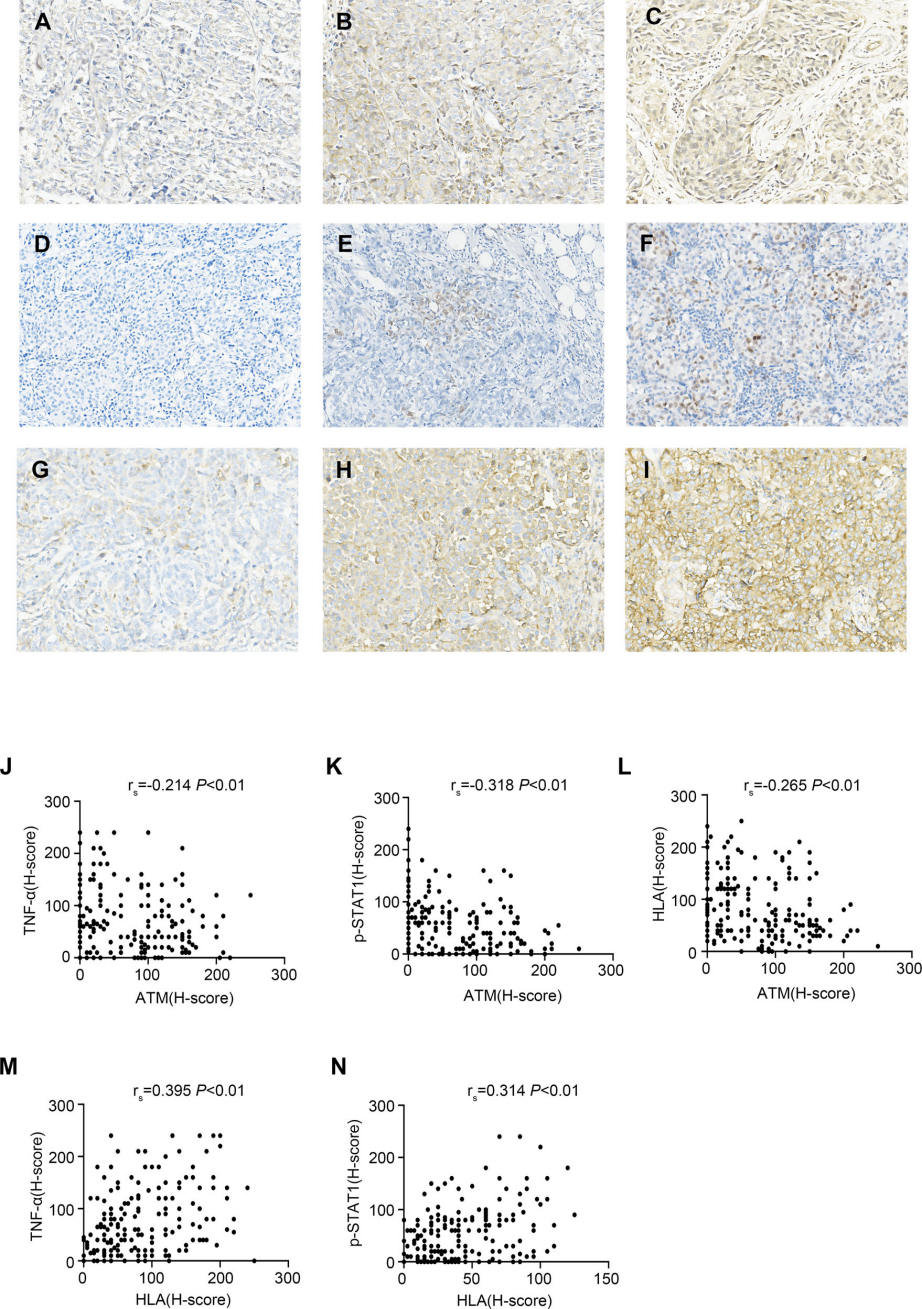
The expression of ATM was negatively correlated with TNF-α, p-STAT1 and MHC-I in TNBC patients. **A**–**I** Representative images of immunohistochemical staining of all molecules expressed in TNBC tissues in this study (200 ×). **A** TNF-α low (H-score: 20); (**B**) TNF-α low (H-score: 100); (**C**) TNF-α high (H-score: 240); (**D**) p-STAT1 low (H-score: 0); (**E**) p-STAT1 high (H-score: 80); (**F**) p-STAT1 high(H-score: 180); (**G**) HLA low (H-score: 30); (**H**) HLA high (H-score: 120); (**I**) HLA high (H-score: 210). **J** Correlation of ATM expression with TNF-α expression. **K** Correlation of ATM expression with p-STAT1 expression. **L** Correlation of ATM expression with HLA expression. **M** Correlation of TNF-α expression with HLA expression. **N** Correlation of p-STAT1 expression with HLA expression.

In order to analyze the correlation between levels of ATM with TNF-α, p-STAT1 and HLA in clinical specimens, we evaluated the ATM, TNF-α, p-STAT1 and HLA immunohistochemical scores of 191 TNBC patients. Based on raw scores obtained from IHC of 191 TNBC patients (Fig. 6A–I), our data showed that ATM H-core was correlated with TNF-α H-core (*r*_s_ = −0.214, *P* = 0.003), p-STAT1 H-core (*r*_s_ = −0.318, *P* < 0.001) and HLA H-core (*r*_s_ = −0.265, *P* < 0.001) (Fig. 6J–L). Additionally, HLA H-core showed a positive correlation with both TNF-α H-core (*r*_s_ = 0.395, *P* < 0.001) and p-STAT1 H-core (*r*_s_ = 0.314, *P* < 0.001) (Fig. 6M, N).

We analyzed triple-negative breast cancer samples with different pathological stages (II/III) and histological grades (1/2/3). ATM expression showed a consistent negative correlation with TNF-α, p-STAT1, and HLA, whereas HLA showed a stable positive correlation with both TNF-α and p-STAT1. These associations remained robust across all subgroups (Table S4–S7).

### ATM inhibition delays tumor growth and sensitizes tumors to PD-1 blockade and radiotherapy

**Fig. 7:**
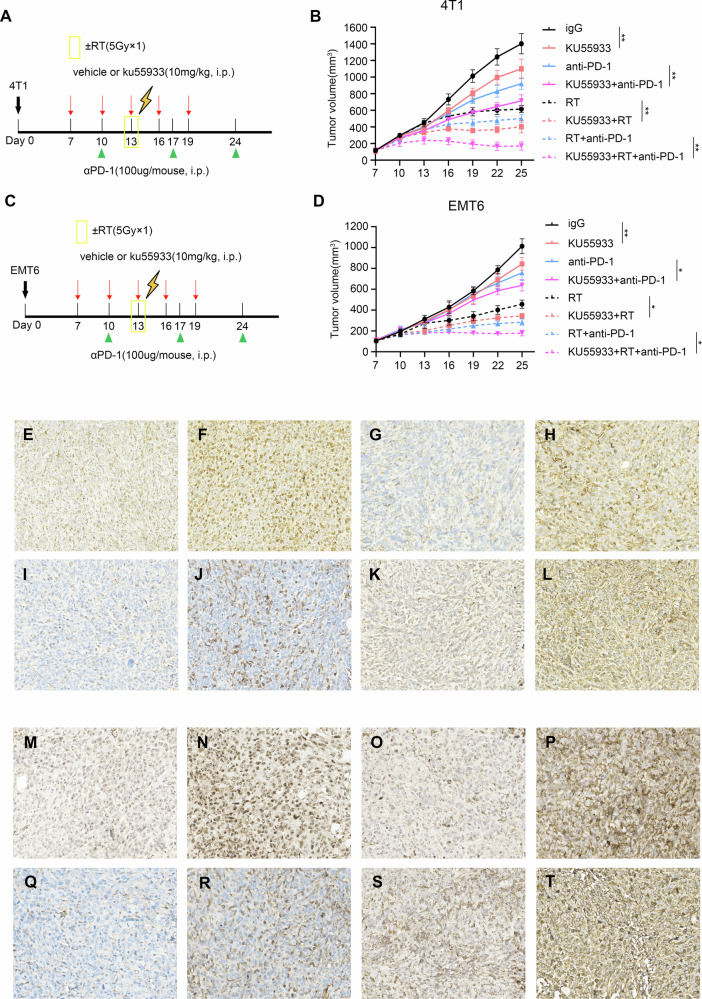
ATM inhibition delays tumor growth and sensitizes tumors to PD-1 blockade and radiotherapy. **A**, **B** 1 × 10^6^ 4T1 cells were orthotopically implanted into the right fourth mammary fat pads of 6–8-week-old female BALB/c mice. When the tumor volume reached about 100 mm^3^, mice received a 10 mg/kg dose of KU55933 or placebo via intraperitoneal injection every three days. Mice received intraperitoneal injections of 100 μg of anti-PD-1 (clone 29 F.1A12™; Bio X Cell) per injection or its isotype control (clone 2A3; Bio X Cell) on days 10, 17and 24. Radiation therapy was administered when the tumor volume of the mice reached approximately 300 mm^3^ (*n* = 5–7/group). **A** Treatment regimen and (**B**) mean tumor volume curves are shown. **C**, **D** 1 × 10^6^ EMT6 cells were orthotopically implanted into the right fourth mammary fat pads of 6–8-week-old female BALB/c mice and subjected to the identical treatment regimen. **C** Treatment regimen and (**D**) mean tumor volume curves are shown. *P < 0.05; **P < 0.01; ns, not significant, *P* > 0.05, by unpaired *t* test (**B**, **D**). **E**–**L** Representative images of immunohistochemical staining of all molecules expressed in 4T1 murine mammary tumor tissues (200 ×). (**E**) p-Atm low (H-score: 40); (**F**) p-Atm high (H-score: 110); (**G**) Tnf-α low (H-score: 30); (**H**) Tnf-α high (H-score: 120); (**I**) p-Stat1 low (H-score: 20); (**J**) p-Stat1 high (H-score: 220); (**K**) H-2 low (H-score: 40); (**L**) H-2 high (H-score: 120). **M**–**T** Representative images of immunohistochemical staining of all molecules expressed in EMT6 murine mammary tumor tissues (200 ×). **M** p-Atm low (H-score: 30); (**N**) p-Atm high (H-score: 120); (**O**) Tnf-α low (H-score: 25); (**P**) Tnf-α high (H-score: 180); (**Q**) p-Stat1 low (H-score: 20); (**R**) p-Stat1 high (H-score: 140); (**S**) H-2 low (H-score: 65); (**T**) H-2 high (H-score: 220).

In our previous work, we demonstrated that ATM knockdown or inhibition did not affect tumor growth in nude mice [33]. In this study, we further examined the effect of ATM on tumor growth and intratumoral CD8^+^ T cell infiltration in BALB/C mice. We inoculated 4T1 cells and EMT6 cells into the fat pad of BALB/C mice, followed by intraperitoneal injection of KU55933 and/or PD-1 neutralizing antibody and/or tumor-directed radiotherapy, as shown (Fig. 7A, C). Tumor growth was recorded every 2–3 days after the appearance of macroscopic tumors. The results showed that ATM inhibition not only significantly delayed tumor growth, but also effectively enhanced the therapeutic effect of anti-PD-1 therapy and radiotherapy (Fig. 7B, D). This suggests that the inhibitory effect of ATM deficiency on tumor growth is dependent on the soundness of the immune system.

As expected, ATM inhibition was most potent when it was combined with anti-PD-1 therapy and radiotherapy, with a limited prolongation of host survival (Fig. S3A, B). We observed that triple therapy (comprising Atm inhibitor therapy, anti-PD-1 therapy, and radiotherapy) resulted in a significant reduction in tumor weight, as compared with ATM inhibitor alone (Fig. S3C, D).

Subsequently, we fixed part of the tumor tissue and performed immunohistochemical staining (Fig. 7E–T). In addition, our data demonstrated that in 4T1 mice, the expression of p-Atm (S1981) was negatively correlated with Tnf-α (*r*_s_ = −0.313, *P* = 0.049), p-Stat1 (Tyr701) (*r*_s_ = −0.368, *P* = 0.019) and H-2 (*r*_s_ = −0.324, *P* = 0.041), and the expression of H-2 expression was positively correlated with Tnf-α (*r*_s_ = 0.485, *P* = 0.002) and p-Stat1 (Tyr701) (*r*_s_ = 0.431, *P* = 0.006) (Fig. S3E-I). Similarly in EMT6 mice, P-ATM (S1981) expression exhibited negative correlations with TNF-α (*r*_s_ = −0.403, *P* = 0.01), P-STAT1 (Tyr701) (*r*_s_ = −0.380, *P* = 0.016), and H-2 (*r*_s_ = −0.350, *P* = 0.027), and the expression of H-2 expression displayed positive correlations with TNF-α (*r*_s_ = 0.324, *P* = 0.042) and P-STAT1 (Tyr701) (*r*_s_ = 0.402, *P* = 0.01) (Fig. S3J–N).

These results suggest that inhibition of ATM increases the expression of MHC-I on the tumor surface, leading to a significant increase in CD8^+^ T cell infiltration, which subsequently inhibits tumor growth. This points to a potential role for ATM in shaping the immune microenvironment.

## Discussion

TNBC, as an aggressive subtype of breast cancer, has high invasiveness, recurrence rate and poor prognosis, and is not sensitive to endocrine therapy and anti-HER2 targeted therapy [37]. In recent years, immunotherapy has made a breakthrough in the treatment of triple-negative breast cancer, providing new treatment options for patients [38]. However, because TNBC is often classified as a “cold” tumor that poses challenges for immune cell infiltration, the application of ICB therapy for TNBC is still limited, and only a small proportion of TNBC patients can benefit from anti-PD-1/PD-L1 therapy [39, 40]. The diversity of immune evasion mechanisms remains a critical barrier to converting non-responsive “cold” tumors into responsive “hot” tumors. Therefore, exploring the mechanisms underlying this shift could provide important insights into the design of effective cancer treatment strategies.

In our study, we found that high expression of ATM was significantly associated with reduced TILs, downregulation of HLA-class I molecules, and insufficient CD8^+^ T cell infiltration, suggesting that ATM may drive immune escape in TNBC by inhibiting antigen presentation and immune cell recruitment. This finding is consistent with previous studies showing that DDR pathway defects such as ATM loss of function may enhance immunogenicity by increasing tumor mutational burden and neoantigen generation [25, 27, 28]. In addition, ATM inhibition could reverse the immune “cold phenotype” of tumor cells by activating the TNF-α/STAT1 signaling pathway and up-regulating the expression of MHC-I molecules. This mechanism provides direct evidence that ATM-targeted interventions reshape the immune microenvironment and explains the potential cause of increased sensitivity to ICI therapy in TNBC patients with low ATM expression. However, whether ATM inhibition affects other immunosuppressive cells, such as Tregs or MDSCs, remains to be further explored.

MHC-I molecules are distributed on the surface of all eukaryotic nucleated cells and consist of heavy alpha and light chains (β2m) and antigenic peptides of 9–10 amino acids [41]. The genes encoding the heavy chain of human MHC-I molecules are mainly composed of human leukocyte antigens HLA-A, HLA-B and HLA-C [42]. As an important hub in the immune system, the core function of MHC-I molecules is to display endogenous peptides to the T cell receptor (TCR) on the surface of CD8^+^ T cells. This process is a key step to trigger the activation of CD8^+^ T cells and thus endow them with cytotoxic potential [43]. This mechanism ensures that the immune system can precisely recognize and eliminate abnormal cells in the body, thereby maintaining the immune balance of the body and anti-tumor immune surveillance. In this study, we provide the first evidence that inhibition of ATM activation increases tumor cell surface antigen presentation by up-regulating the expression of MHC-I molecules, so that more CD8^+^ T cells can recognize and attack tumor cells, and regulate the function of other immune cells by secreting cytokines, and enhance the tumor immunity “heat” of TNBC. And further enhance the efficacy of anti-tumor immunotherapy. In addition, HLA up-regulation mediated by the TNF-α/STAT1 pathway suggests that ATM inhibition may overcome ICI resistance due to MHC-I depletion, providing a new idea for the treatment dilemma of patients with PD-L1-positive but HLA-low expression.

Previous studies have shown that tumor responsiveness to ICB therapy is regulated by multiple factors, including the abundance of PD-L1 expression, the level of tumor mutation burden (TMB), and the “heat” of tumor immune microenvironment (TIME) [44–46]. However, the anti-tumor response rate of ICB monotherapy remains relatively low, prompting a shift towards combination therapies as a common clinical approach.

The ATM gene was first identified in patients with ataxia telangiectasia, and these individuals typically exhibit increased susceptibility to cancer and sensitivity to ionizing radiation [20]. ATM plays an important role in DDR and cell cycle regulation [16, 17]. Alterations in the DDR pathway not only lead to genomic instability and neoantigen generation, but also affect the tumor immune microenvironment through mechanisms such as upregulation of PD-L1 [27, 28, 47]. Studies have shown that radiotherapy can synergize with immunotherapy by enhancing the release and presentation of tumor antigens, activating immune cells, increasing the density of tumor-infiltrating lymphocytes, and remodeling the tumor microenvironment, thereby transforming “cold” tumors into “hot” tumors [48, 49]. Thus, on the one hand, ATM deficiency can enhance radiosensitivity by hindering DNA double-strand break repair [50, 51]. On the other hand, inhibition of ATM can up-regulate the expression of HLA through c-Jun/TNF-α/p-STAT1, thereby enhancing CD8^+^ T cells infiltration and increasing the immune “heat” in TNBC. This dual effect of “radiosensitization and immune activation” further strengthens the reversal of the immune “cold phenotype” of tumor cells. However, whether the toxicity of this triple therapy (ATM inhibitor + radiotherapy + ICI) can be controlled and whether it can accurately screen the patients who can benefit from it still need to be further explored.

**Fig. 8:**
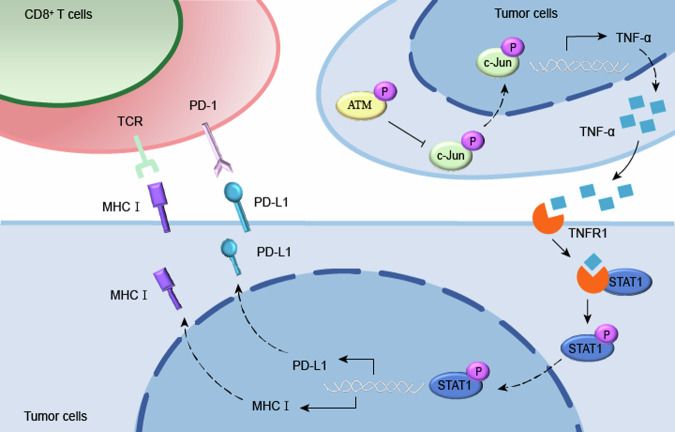
Summary of the role of ATM downregulating MHC-I expression by inactivating the c-Jun/TNF-α/p-STAT1 signaling axis in TNBC. The Schematic model summarizes the promotion effect of ATM/c-Jun/TNF-α/p-STAT1 signaling axis on MHC-I expression in TNBC. ATM inhibition upregulates TNF-α expression at both transcriptional and translational levels by promoting c-Jun phosphorylation and nuclear translocation, thereby enhancing its binding to the TNF-α promoter region. The elevated TNF-α subsequently facilitates STAT1 phosphorylation through TNFR1-STAT1 interaction, ultimately mediating increased expression of both MHC class I and PD-L1 molecules. The up-regulation of PD-L1 molecules is the result of the regulation of ATM/JNK/c-Jun/TNF-α signaling axis confirmed by our previous study [33].

In conclusion, our results provide insight into the molecular mechanism by which ATM negatively regulates MHC-I expression through the TNF-α/p-STAT1 signaling axis (Fig. 8). ATM plays a key role in CD8^+^ T cell-mediated tumor immune regulation. Our results suggest that ATM negative regulation of MHC-I is a key mechanism of immune escape in triple-negative breast cancer. ATM may be a promising marker for anti-PD-L1 therapy and radiation therapy. The efficacy of ICB in TNBC was improved by ATM inhibition and further augmented by radiation, highlighting the combination of ATM inhibition with ICB and radiation as an effective treatment strategy for breast cancer.

## Supplementary information

Supplementary Materials

western blot raw data

## Data Availability

The data supporting the findings of this study are available from the corresponding author upon reasonable request.
