# Supplementary material for: Inhibition of ATM enhances the immunogenicity of triple-negative breast cancer by promoting MHC-I expression
Source: Cell Death Dis. 2025 Aug 18;16(1):624. doi: 10.1038/s41419-025-07944-y (PMC12361503; doi:10.1038/s41419-025-07944-y)
Supplement: Supplementary file 1 — Supplementary Materials [file 41419_2025_7944_MOESM1_ESM.pdf]

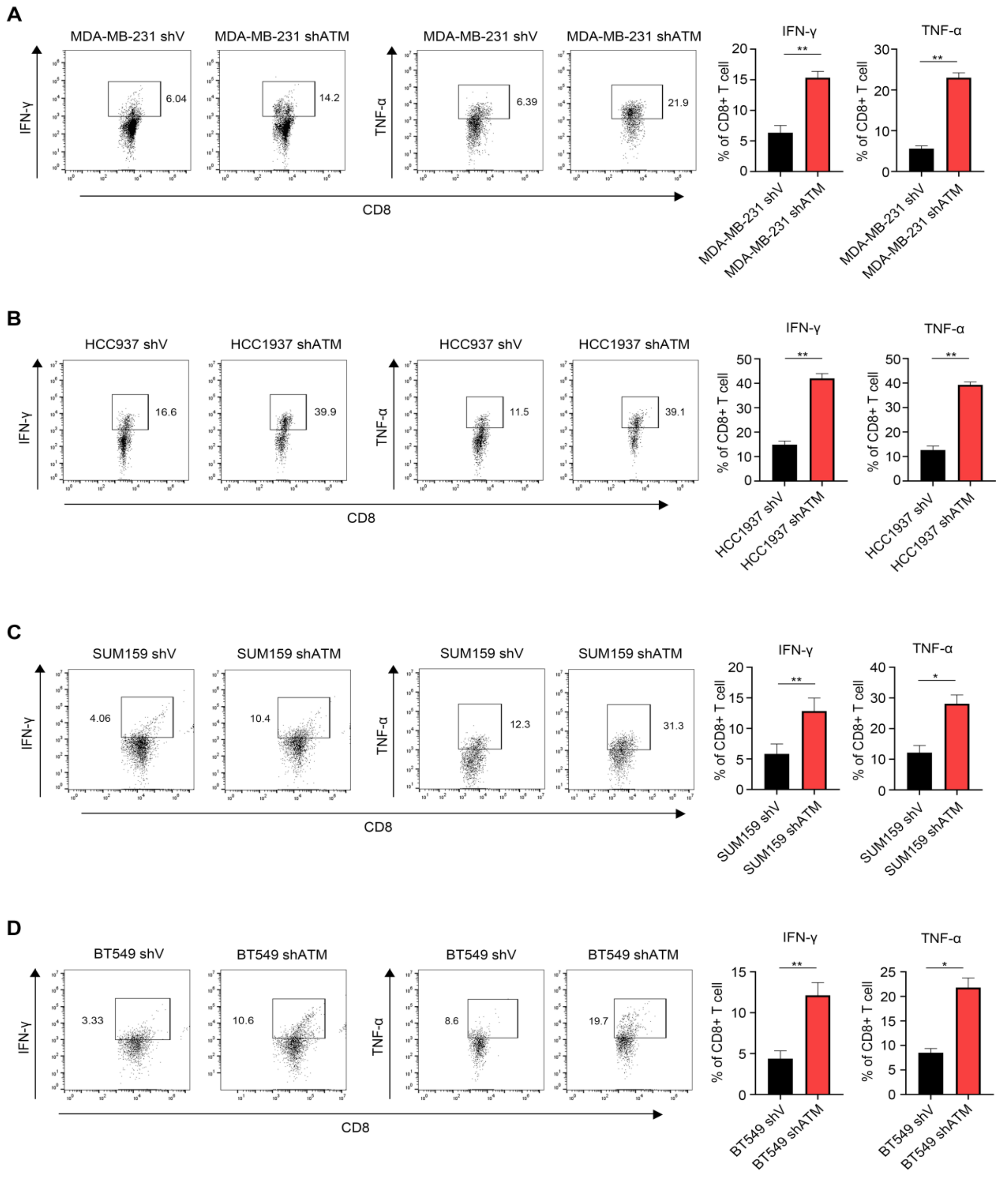

Supplementary Fig.1 ATM knockdown is expected to improve the immune "heat" of TNBC

(A-D)Representative dot plots (left) and statistical analysis(right) of IFN-γ^+ CD8$^+$ T cells and TNF-α^+ CD8$^+$ T cells cocultured with the indicated cell line. Error bars represent mean ± SD. Two-tailed Student’s t-test was used for statistical analysis. **P < 0.01; *0.01<P < 0.05; ns, not significant, P > 0.05.

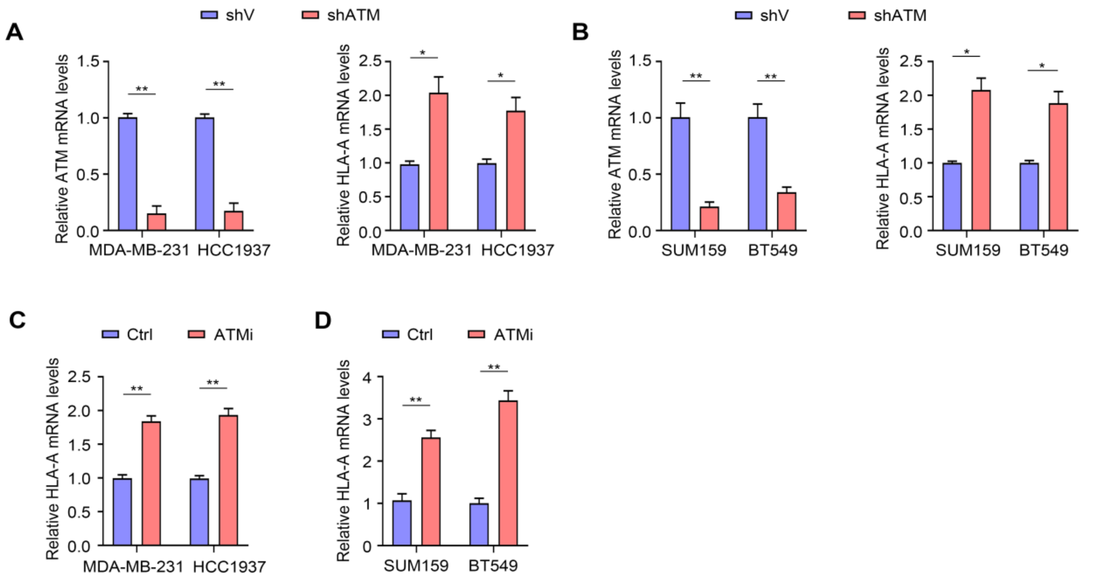

Supplementary Fig.2 ATM silencing and inhibition upregulate HLA-A mRNA expression in TNBC cell lines

(A and B) qRT-PCR analyses showed the effect of silencing ATM on the mRNA levels of ATM and HLA-A in MDA-MB-231, HCC1937, SUM159 and BT549 cells with indicated treatments. (C and D) qRT-PCR analyses showed the effect of ATM inhibition on the mRNA levels of HLA-A in MDA-MB-231, HCC1937, SUM159 and BT549 cells with indicated treatments. Error bars represent mean ± SD. Two-tailed Student's t-test was used for statistical analysis. $^{**}P < 0.01$; $^{*}0.01 < P < 0.05$; ns, not significant, $P > 0.05$.

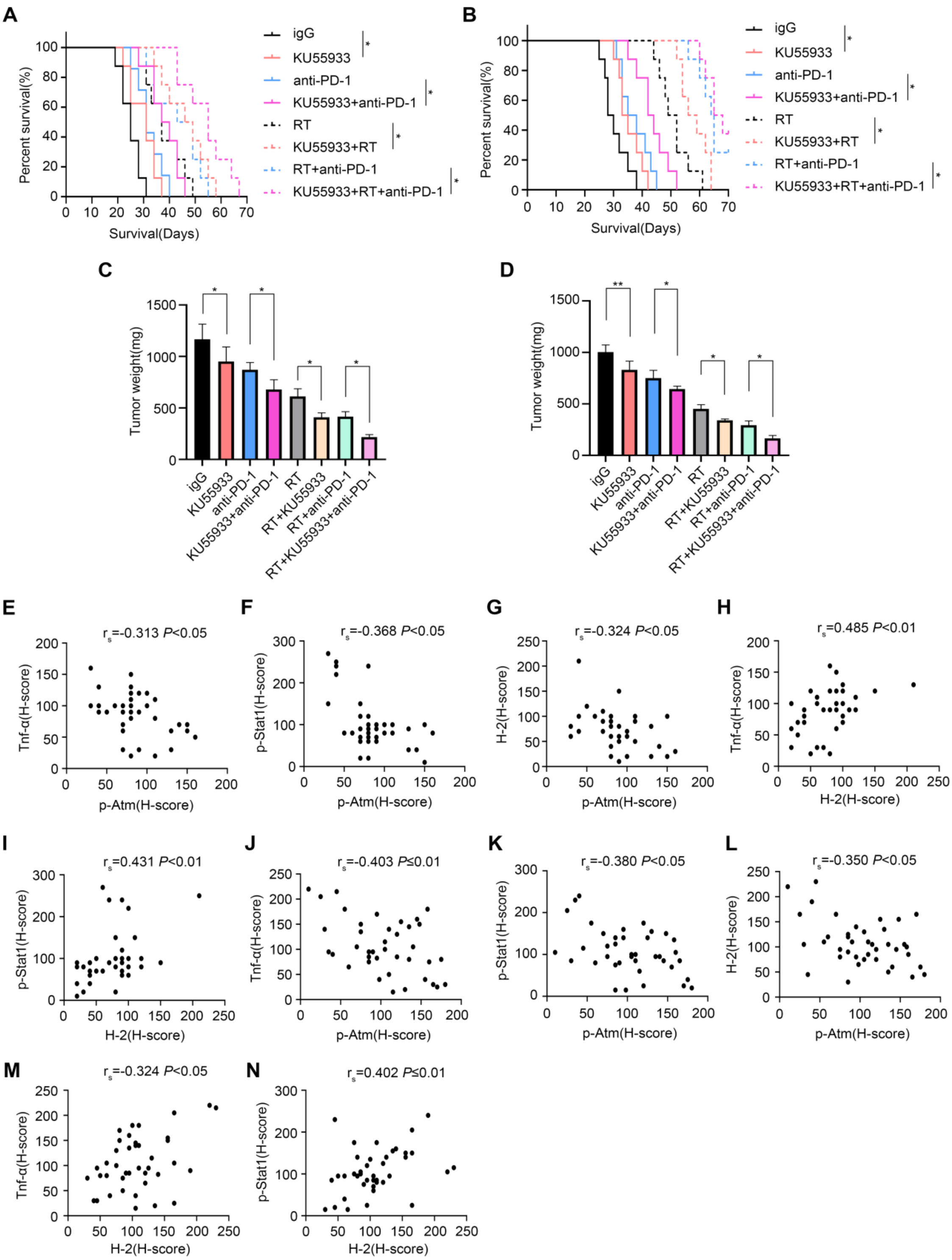
A
Percent survival(%)
Survival(Days)
igG
KU55933
anti-PD-1
KU55933+anti-PD-1
RT
KU55933+RT
RT+anti-PD-1
KU55933+RT+anti-PD-1
B
Percent survival(%)
Survival(Days)
igG
KU55933
anti-PD-1
KU55933+anti-PD-1
RT
KU55933+RT
RT+anti-PD-1
KU55933+RT+anti-PD-1
C
Tumor weight(mg)
igG
KU55933
anti-PD-1
KU55933+anti-PD-1
RT
RT+KU55933
RT+anti-PD-1
RT+KU55933+anti-PD-1
D
Tumor weight(mg)
igG
KU55933
anti-PD-1
KU55933+anti-PD-1
RT
RT+KU55933
RT+anti-PD-1
RT+KU55933+anti-PD-1
E
rs=-0.313 P<0.05
Tnf-α(H-score)
p-Atm(H-score)
F
rs=-0.368 P<0.05
p-Stat1(H-score)
p-Atm(H-score)
G
rs=-0.324 P<0.05
H-2(H-score)
p-Atm(H-score)
H
rs=0.485 P<0.01
Tnf-α(H-score)
H-2(H-score)
I
rs=0.431 P<0.01
p-Stat1(H-score)
H-2(H-score)
J
rs=-0.403 P≤0.01
Tnf-α(H-score)
p-Atm(H-score)
K
rs=-0.380 P<0.05
p-Stat1(H-score)
p-Atm(H-score)
L
rs=-0.350 P<0.05
H-2(H-score)
p-Atm(H-score)
M
rs=-0.324 P<0.05
Tnf-α(H-score)
H-2(H-score)
N
rs=0.402 P≤0.01
p-Stat1(H-score)
H-2(H-score)

Supplementary Fig.3 ATM inhibition delays tumor growth and sensitizes tumors to PD-1 blockade and radiotherapy

(A and C) 1×10^6 4T1 cells were orthotopically implanted into the right fourth mammary fat pads of 6-8-week-old female BALB/c mice. (A) mouse survival data and (C) mouse tumor weight are shown. (B and D) 1×10^6 EMT6 cells were orthotopically implanted into the right fourth mammary fat pads of 6-8-week-old female BALB/c mice and subjected to the identical treatment regimen. (B) mouse survival data and (D) mouse tumor weight are shown. *$P < 0.05$; **$P < 0.01$; ns, not significant, $P > 0.05$, by log-rank test (A and B) or unpaired two-tailed t test (C and D). (E-I) In the 4T1 model: (E) Correlation of p-Atm expression with Tnf-α expression. (F) Correlation of p-Atm expression with p-Stat1 expression. (G) Correlation of p-Atm expression with H-2 expression. (H) Correlation of H-2 expression with Tnf-α expression. (I) Correlation of H-2 expression with p-Stat1 expression. (J-N) In the EMT6 model: (J) Correlation of p-Atm expression with Tnf-α expression. (K) Correlation of p-Atm expression with p-Stat1 expression. (L) Correlation of p-Atm expression with H-2 expression. (M) Correlation of H-2 expression with Tnf-α expression. (N) Correlation of H-2 expression with p-Stat1 expression. (RT, radiation therapy).

Supplementary Table 1 The sequences of siRNAs used for gene silencing

Gene	Sense sequences
sh ATM	5′-CGGCATTCAGATTCCAAACAA-3′
sh TNF	5′-ctGTAGCCCATGTTGTAGCAA-3′
si STAT1	5′-GGAAGAUUUACAAGAUGAATT-3′
si c-Jun	5′-CCAACATGCTCAGGGAACA-3′

Supplementary Table 2 The primer sequences for this study

Gene	Primer sequences
ATM	sense primer 5′-GGCTATTCAGTGTGCGAGACA-3′
	antisense primer 5′-TGGCTCCTTTCGGATGATGGA-3′
TNF-α	sense primer 5′-CCTCTCTCTAATCAGCCCTCTG-3′
	antisense primer 5′-GAGGACCTGGGAGTAGATGAG-3′
HLA-A	sense primer 5′-CGTCGCGGTCGCTGTT-3′
	Antisense primer 5′-AGAAATACCTCATGGAGTGGGAG-3′
B2M	sense primer 5′-CCACTGAAAAAGATGAGTATGCCT-3′
	antisense primer 5′-CCAATCCAAATGCGGCATCTTCA-3′
TAPBP	sense primer 5′-GAGCCTGTTCTCATCACCATGG-3′
	antisense primer 5′-GTAGGCAAAGCTCAAGTCCAGC-3′
ERAP1	sense primer 5′-GTCTGTCAGTGTGACCCATCCT-3′
	antisense primer 5′-CTGAGCAGGATTTTCCACAGGTG-3′
TAP1	sense primer 5′-GCAGTCAACTCCTGGACCACTA-3′
	antisense primer 5′-CAAGGTTCCCACTGCTTACAGC-3′
TAP2	sense primer 5′-ATGCCCTTCACAATAGCAGCGG-3′
	antisense primer 5′-CCAAAACTGCGAACGGTCTGCA-3′
NLRC5	sense primer 5′-AGTGGCTCTTCCGCTTGGACAT-3′
	antisense primer 5′-CGGAACCCTAAGAACTTGGCTG-3′
GAPDH	sense primer 5′-CTGGGCTACACTGAGCACC-3′
	antisense primer 5′-AAGTGGTCGTTGAGGGCAATG-3′

Supplementary Table 3 Raw data from all 191 patients with triple-negative breast cancer

Cases	TNM staging	Histologic grade	Histological type	Age	KI67(%)	TILs(%)	ATM（H-score）	TNFα（H-score）	HLA（H-score）	p-STAT1（H-score）	CD8（cells/HPF）
1	$pT2N2aM_0$	II	Invasive ductal carcinoma (IDC)	46	80	5	60	20	100	80	55
2	$pT2N2aM_0$	III	IDC	43	35	10	0	140	240	180	139
3	$pT1N3aM_0$	III	IDC	59	70	5	60	120	110	100	6
4	$pT2N2aM_0$	III	IDC	60	80	30	100	20	50	50	96
5	$pT2N3aM_0$	II	IDC	51	80	30	0	150	80	140	78
6	$pT1cN3aM_0$	II	IDC	59	70	10	20	160	160	90	30
7	$pT2N3aM_0$	II	IDC	52	25	70	60	120	70	75	50
8	$pT2N2aM_0$	III	IDC	57	90	20	180	80	30	0	16
9	$pT1cN2aM_0$	II	IDC	63	35	10	25	240	200	110	17
10	$pT2N3aM_0$	III	IDC	61	85	5	90	100	140	0	80
11	$pT2N3aM_0$	III	IDC	71	60	30	150	40	170	40	10
12	$pT2N2aM_0$	III	IDC	53	60	20	0	160	150	140	15
13	$pT3N2aM_0$	II	IDC	35	70	10	0	50	30	130	30
14	$pT2N2aM_0$	II	IDC	44	80	5	210	10	90	10	75
15	$pT2N2aM_0$	III	IDC	73	80	40	0	220	200	220	58
16	$pT2N3aM_0$	III	IDC	32	90	10	30	180	160	80	64
17	$pT1cN3aM_0$		Apocrine adenocarcinoma	51	60	5	60	120	120	80	32

18	pT1cN3aMx	III	IDC	48	90	30	0	20	140	240	48
19	pT4bN3aM$_0$	III	IDC	83	70	20	160	30	150	30	77
20	pT2N3aM$_0$	III	IDC	42	70	20	20	150	120	180	20
21	pT1cN3aM$_0$	II	IDC	74	70	5	60	40	40	150	40
22	pT2N3aM$_0$	III	IDC	48	85	10	130	80	140	0	170
23	pT2N3aM$_0$	II	IDC	59	30	5	150	70	50	80	10
24	pT1cN3aM$_0$	II	IDC with apocrine metaplasia	53	70	5	30	210	80	60	20
25	pT2N3aM$_0$	II	IDC	42	80	30	20	210	90	0	100
26	pT2N3aM$_0$	III	IDC	57	40	30	140	40	70	160	30
27	pT1cN3aM$_0$	III	IDC	70	70	20	30	180	90	0	75
28	pT2N3aM$_0$	III	IDC	65	70	60	30	120	130	20	10
29	pT2N3aM$_0$	III	IDC	53	70	10	150	100	140	90	5
30	pT1cN3aM$_0$	III	IDC	53	80	30	0	240	170	240	60
31	pT2N3aM$_0$	III	IDC	48	50	50	0	180	90	30	150
32	pT2N3aM$_0$	III	IDC with apocrine metaplasia	51	20	5	120	140	140	80	10
33	pT3N3aM$_0$		IDC with apocrine metaplasia	32	20	5	150	40	60	150	15
34	pT2N3cM$_0$	II	IDC	72	60	5	100	100	120	60	25
35	pT3N3aM$_0$		IDC	64	45	5	100	20	70	60	30

36	pT3N3aM$_0$	II	IDC	47	40	5	120	20	150	80	40
37	pT2N3aM$_0$	III	IDC	69	80	5	110	80	180	160	50
38	pT2N3aM$_0$		invasive lobular carciNoma	56	50	50	30	140	110	0	10
39	pT2N3aM$_0$	III	IDC	47	70	30	20	180	40	30	70
40	pT2N3aM$_0$	III	IDC with apocrine metaplasia	64	25	10	0	30	50	140	27
41	pT3N3aM$_0$	III	IDC	45	25	10	150	160	190	20	28
42	pT2N3aM$_0$	II	IDC	53	60	5	0	40	20	80	50
43	pT2N3aM$_0$	III	IDC	55	70	10	20	180	160	90	20
44	pT2N3aM$_0$	II	IDC	52	80	20	5	80	220	70	140
45	pT2N3aM$_0$	III	IDC	44	10	5	120	80	190	10	10
46	ypT3N2M$_0$		IDC	65	50	5	15	40	170	15	15
47	pT2N2aM$_0$	III	IDC	30	60	10	90	160	30	0	20
48	pT3N2aM$_0$	III	IDC	65	30	5	150	10	100	20	10
49	pT3N1aM$_0$	III	IDC	50	20	5	0	180	20	50	30
50	pT2N3aM$_0$	III	IDC	61	80	5	80	50	10	10	40
51	pT2N2aM$_0$	III	IDC	59	30	5	120	90	60	0	150
52	pT2N2aM$_0$	III	IDC	51	35	30	20	50	120	70	400
53	pT2N2aM$_0$	III	IDC	70	90	10	160	40	40	60	60
54	pT3N2aM$_0$	II	IDC	48	25	30	90	20	90	30	180
55	pT2N2aM$_0$	II	IDC	38	35	10	30	210	140	120	20
56	pT2N2aM$_0$	III	IDC	50	65	20	120	110	160	30	30

57	$pT2N3aM_0$	II	Squamous cell carciNoma	55	90	10	0	160	150	30	200
58	$pT3N3aM_0$	II	IDC	55	60	30	50	160	70	80	69
59	$pT2N2aM_0$	III	IDC with apocrine metaplasia	56	40	5	100	240	130	5	25
60	$pT2N2aM_0$	III	IDC	50	35	10	15	10	50	60	400
61	$pT2N2aM_0$	II	IDC with apocrine metaplasia	64	15	5	150	140	170	0	50
62	$pT2N2aM_0$	III	IDC	63	30	5	50	240	190	60	5
63	$pT2N2aM_0$	II	IDC	29	70	50	0	100	140	70	200
64	$pT2N2aM_0$	II	IDC	63	70	30	40	180	110	60	5
65	$pT2N3aM_0$	III	IDC	53	50	30	0	50	80	5	210
66	$pT1N3aM_0$	II	IDC	38	70	5	50	240	40	10	200
67	$pT1cN3aM_0$	II	IDC	70	50	5	0	80	140	20	100
68	$pT1cN2aM_0$	II	IDC	52	5	20	90	140	50	30	80
69	$pT2N2aM_0$	III	IDC	38	40	70	30	210	180	140	250
70	$pT1cN2aM_0$	II	IDC	68	25	30	0	40	160	10	100
71	$pT2N2aM_0$	II	IDC	49	60	30	150	210	50	20	50
72	$pT2N2aM_0$	II	IDC	53	35	10	30	120	210	120	100
73	$pT1cN2aM_0$	II	IDC	42	30	20	210	80	40	20	300
74	$pT2N2aM_0$	III	IDC	49	80	10	250	120	10	10	150
75	$pT3N2aM_0$	II	IDC	66	90	5	150	210	90	20	70

76	pT2N2aM_0	II	IDC	43	50	40	30	100	170	130	200
77	pT2N2aM_0	II	IDC	69	35	10	200	120	20	10	50
78	pT2N2aM_0		IDC	56	80	5	200	60	80	0	10
79	pT2N2aM_0	III	IDC	50	50	60	0	140	210	160	100
80	pT2N2aM_0	III	IDC	54	50	5	100	140	190	70	15
81	pT2N2aM_0	II	IDC	44	70	30	160	110	60	70	5
82	pT2N3aM_0	III	IDC	56	80	30	100	120	80	50	20
83	pT1cN2aM_0	II	IDC	43	45	5	30	60	120	60	5
84	pT1cN3aM_0	II	IDC	51	70	20	60	100	40	70	150
85	pT1cN2aM_0	II	IDC	46	20	20	60	10	30	60	20
86	pT2N3aM_0	III	IDC	50	80	30	140	150	50	90	80
87	pT2N0M_0	III	IDC	61	60	20	70	40	60	85	75
88	pT2N0M_0	III	IDC	64	60	50	0	50	50	80	25
89	pT2N0M_0	III	IDC	41	60	5	80	35	5	30	5
90	pT2N0M_0	II	IDC	60	60	60	15	150	120	100	0
91	pT2N0M_0	II	IDC	33	50	5	50	0	0	0	100
92	pT2N0M_0	III	IDC	50	90	10	95	10	40	20	30
93	pT2N0M_0	III	IDC	63	40	20	40	90	120	45	110
94	pT2N0M_0	III	IDC	50	60	30	120	20	70	20	20
95	pT1cN1aM_0	II	IDC	60	15	50	0	180	100	145	75
96	pT2N1aM_0	III	IDC	34	80	5	120	40	70	5	20
97	pT2N1miM_0	III	IDC	45	50	60	220	0	40	55	0
98	pT2N1miM_0	III	IDC	45	60	10	35	70	40	0	20

99	pT2N1aM_0	III	IDC	49	90	30	160	50	50	5	25
100	pT1cN1aM_0	III	IDC	50	60	0	80	35	40	10	15
101	pT2N1aM_0	III	IDC	63	90	80	130	40	70	0	25
102	pT2N0M_0	II	IDC	52	70	20	150	40	55	20	15
103	pT1cN1aM_0	II	IDC	56	20	5	170	20	40	20	75
104	pT2N0M_0	II	IDC	55	45	10	115	0	0	40	0
105	pT2N0M_0	II	IDC	62	30	20	150	45	100	80	0
106	pT2N1aM_0	III	IDC	47	40	5	120	20	15	40	40
107	pT2N0M_0	III	IDC	62	70	20	165	25	45	20	20
108	pT2N0M_0	III	IDC	53	85	50	200	40	20	45	225
109	pT2N0M_0	III	IDC	75	45	40	40	65	140	160	60
110	pT2N0M_0	II	IDC	55	80	40	160	10	30	5	65
111	pT2N0M_0	II	IDC	46	70	10	90	40	0	0	10
112	pT1cN1aM_0	II	IDC	57	20	0	20	0	40	60	60
113	ypT2N1aM_0		IDC	47	60	40	75	25	5	10	125
114	pT2N1aM_0	II	IDC	45	35	5	70	150	140	0	0
115	pT2N0M_0	III	IDC	33	60	5	50	85	75	0	5
116	pT2N0M_0	III	IDC	61	85	30	110	0	30	40	25
117	pT1cN1aM_0	II	IDC	46	70	80	0	80	55	20	120
118	pT2N0M_0	III	IDC	46	60	5	110	0	20	10	35
119	pT2N0M_0	II	IDC	65	80	5	80	45	0	0	20
120	pT2N0M_0	II	IDC	42	70	40	15	60	35	100	80
121	pT2N0M_0	III	IDC	40	80	20	130	20	40	0	80

122	pT1cN1miM$_0$	II	IDC with apocrine metaplasia	74	15	20	30	0	40	85	0
123	pT2N0M$_0$	III	IDC	41	90	40	35	55	220	30	300
124	pT1cN1aM$_0$	III	IDC	66	50	5	180	60	60	5	55
125	pT2N0M$_0$	III	IDC	48	80	50	70	40	180	10	25
126	pT2N1aM$_0$	II	IDC	47	80	20	110	40	175	95	45
127	pT2N0M$_0$	II	IDC	58	50	20	90	0	30	0	5
128	pT2N0M$_0$	III	IDC	64	70	40	25	65	20	70	80
129	pT2N0M$_0$	II	IDC	41	90	30	95	25	75	5	20
130	pT1cN1aM$_0$	III	IDC	52	90	40	155	10	50	60	45
131	pT2N0M$_0$	III	IDC	48	30	20	135	60	210	20	35
132	pT2N0M$_0$	III	IDC	37	60	10	135	70	50	105	5
133	pT2N0M$_0$	II	IDC	46	90	30	115	50	60	80	10
134	pT2N1miM$_0$	II	IDC	39	80	5	150	35	35	0	35
135	pT2N0M$_0$	III	IDC	38	60	20	45	30	195	120	10
136	pT1cN1aM$_0$	II	IDC	36	70	10	165	90	30	25	5
137	pT2N0(sn)M$_0$	III	IDC	48	80	30	120	0	80	140	35
138	pT2N0M$_0$	III	IDC	70	70	5	20	70	160	0	5
139	pT1cN1aM$_0$		Apocrine adenocarcinoma	60	20	5	5	60	100	85	70
140	pT1cN1aM$_0$	II	IDC	53	80	30	45	10	125	0	5
141	pT2N1aM$_0$	III	IDC	61	80	20	135	15	10	60	0
142	pT2N1aM$_0$	III	IDC	41	85	5	85	0	60	20	0

143	pT2N0M_0	III	IDC	34	80	30	60	20	80	40	165
144	pT2N1aM_0	III	IDC	47	70	20	205	0	30	40	30
145	pT1cN1miM_0	III	IDC	44	40	20	35	200	120	90	80
146	pT2N1aM_0	III	IDC	50	80	20	90	10	55	15	10
147	pT2N0（sn）M_0	III	IDC	60	30	10	95	15	15	35	0
148	pT2N1aM_0	III	IDC	59	90	10	130	100	55	15	40
149	pT2N0M_0	III	IDC	61	80	10	10	95	80	95	70
150	pT2N0M_0		IDC with apocrine metaplasia	53	70	10	0	70	40	10	55
151	pT2N1aM_0	III	Apocrine adenocarcinoma	62	30	40	0	65	170	110	110
152	pT2N0M_0	III	IDC	41	80	50	20	65	50	60	100
153	pT2N1aM_0	III	IDC	41	80	30	20	0	90	80	25
154	pT2N0M_0	III	IDC	62	20	10	0	115	30	30	10
155	pT2N0M_0	III	IDC	46	90	20	40	90	110	15	140
156	pT2N0M_0	III	IDC	57	85	20	60	80	45	0	10
157	pT2N0M_0	III	IDC	51	90	50	0	20	70	60	95
158	pT2N0M_0		Apocrine adenocarcinoma	63	25	30	10	65	20	0	40
159	pT2N1aM_0		invasive lobular carciNoma	70	20	5	10	10	80	70	10
160	pT2N0M_0	II	IDC	58	20	40	20	0	60	0	5

161	pT1cN1aM$_0$	III	IDC	47	70	20	40	65	25	50	5
162	pT2N0M$_0$	III	IDC	42	60	5	30	135	95	60	0
163	pT2N1aM$_0$	III	IDC	37	90	20	0	60	75	60	85
164	ypT2N0M$_0$		IDC	33	30	65	0	20	120	95	110
165	pT2N0M$_0$	III	IDC	54	50	5	15	90	40	55	60
166	pT2N0M$_0$	II	IDC	64	80	40	0	125	80	30	20
167	pT2N1aM$_0$	III	IDC	44	70	10	10	0	15	60	15
168	pT2N0M$_0$	III	IDC	43	80	60	0	50	80	40	20
169	pT1cN1a（sn）M$_0$	III	IDC	36	80	70	25	10	120	85	100
170	pT2N1aM$_0$	II	IDC	45	70	10	110	20	40	10	0
171	pT2N0M$_0$	II	IDC	56	80	10	50	0	250	90	0
172	pT2N0M$_0$	III	IDC	34	90	10	30	110	65	0	35
173	pT2N0M$_0$	III	IDC	56	80	60	0	0	80	80	45
174	pT2N0M$_0$	III	IDC	56	80	10	20	55	130	40	0
175	pTxN0M$_0$	III	IDC	47	60	10	60	100	80	50	165
176	pT2N0（sn）M$_0$	III	IDC	32	80	50	0	50	85	120	95
177	pT2N0M$_0$	III	IDC	58	90	5	90	135	40	75	20
178	pT2N0M$_0$	III	IDC with apocrine metaplasia	50	20	5	20	0	125	70	0
179	pT2N0M$_0$	III	IDC	45	65	10	150	20	20	80	5
180	pT2N0M$_0$	III	IDC	46	80	30	100	40	30	5	15

181	pT2N1aM_0	II	IDC	37	80	5	95	0	0	80	0
182	pT2N0M_0	III	IDC with apocrine metaplasia	55	30	5	90	30	5	60	0
183	pT2N0M_0	II	invasive lobular carciNoma	57	10	10	80	0	20	25	40
184	pT2N1aM_0	III	IDC	43	80	80	20	20	30	10	105
185	pT2N0M_0	III	IDC	66	70	10	100	70	25	0	65
186	pT2N0M_0	II	IDC	38	55	5	150	10	0	15	0
187	pT2N0M_0	II	IDC	74	80	5	155	15	30	40	15
188	pT2N0M_0	III	IDC	55	85	10	100	60	60	65	15
189	pT2N1aM_0	III	IDC	50	60	30	50	0	20	5	5
190	pT1cN M_0	III	IDC	43	85	20	150	35	100	20	20
191	pT2N1aM_0	III	IDC	51	80	30	140	65	30	40	40

Supplementary Table 4 Correlation between ATM and TILs, $CD8^{+}$ T cells, TNF-α, p-STAT1 and HLA in TNBC of different stages

Indicators	ATM[a]					
	stage II (N=105)			stage III(N=86)		
	$M(Q_R)$[d]	r_s	P	$M(Q_R)$[d]	r_s	P
TILs[b]	20(25)	-0.214	0.028	10(25)	-0.281	0.009
CD8+[c]	25(62.5)	-0.231	0.018	50(80)	-0.239	0.027
TNF-α[a]	40(55)	-0.259	0.008	115(110)	-0.229	0.034
p-STAT1[a]	40(62.5)	-0.270	0.005	60(70)	-0.363	0.001
HLA[a]	50(52.5)	-0.332	0.001	110(110)	-0.246	0.022

[a] Caculated by H-Score (Staining intenSity × Percentage of positive cells).

[b] Scored using the 2014 international TILs working group standard.

[c] Average number of positive cells in 10 fields at high magnification (× 400).

[d] Expressed by median (interquartile range).

Supplementary Table 5 Correlation between HLA and TNF-α, p-STAT1 in TNBC of different stages

Indicators	HLA[a]					
	stage II (N=105)			stage III(N=86)		
	$M(Q_R)$[b]	r_s	P	$M(Q_R)$[b]	r_s	P
TNF-α[a]	40(55)	0.291	0.003	115(110)	0.224	0.038
p-STAT1[a]	40(62.5)	0.301	0.002	60(70)	0.258	0.017

[a] Caculated by H-Score (Staining intenSity × Percentage of positive cells).

[b] Expressed by median (interquartile range).

Supplementary Table 6 Correlation between ATM and TILs, CD8+ T cells, TNF-α, p-STAT1 and HLA in TNBC of different grades

Indicators	ATM[a]					
	grade 1/2(N=66)			grade 3(N=113)		
	$M(Q_R)$[d]	r_s	P	$M(Q_R)$[d]	r_s	P
TILs[b]	10(25)	-0.291	0.018	20(20)	-0.189	0.045
CD8+[c]	40(69.75)	-0.259	0.036	30(65)	-0.202	0.032
TNF-α[a]	60(120)	-0.251	0.042	60(90)	-0.218	0.020
p-STAT1[a]	50(70)	-0.343	0.005	45(67.5)	-0.346	< 0.001
HLA[a]	65(86.25)	-0.344	0.005	70(95)	-0.234	0.012

[a] Caculated by H-Score (Staining intenSity × Percentage of positive cells).

[b] Scored using the 2014 international TILs working group standard.

[c] Average number of positive cells in 10 fields at high magnification (× 400).

[d] Expressed by median (interquartile range).

Supplementary Table 7 Correlation between HLA and TNF-α, p-STAT1 in TNBC of different grades

Indicators	HLA[a]					
	grade 1/2(N=66)			grade 3(N=113)		
	$M(Q_R)$[b]	r_s	P	$M(Q_R)$[b]	r_s	P
TNF-α[a]	60(120)	0.427	<0.001	60(90)	0.395	<0.001
p-STAT1[a]	50(70)	0.304	0.013	45(67.5)	0.300	0.001

[a] Caculated by H-Score (Staining intenSity × Percentage of positive cells).

[b] Expressed by median (interquartile range).

Supplementary Materials and Methods

Cell culture and stable cell line establishment

Human TNBC cell lines MDA-MB-231 and HCC1937, and murine TNBC cell line 4T1 were purchased from PROCELL company. Cells were cultured in Dulbecco's modified Eagle's medium (DMEM; VivaCell Biosciences, Shanghai) containing 10% foetal bovine serum (FBS; VivaCell Biosciences) and 1% penicillin streptomycin antibiotic (VivaCell Biosciences). All cells were maintained in a humidified 5% CO2 environment at 37°C. For inhibition studies, ATM inhibitor KU55933 (10μM) (MedChemExpress, HY-12016) and TNF-α neutralizing infliximab (10 μg/ml) (MedChemExpress, HY-P9970) were added into the culture.

In order to construct stable cell lines with knockdown of ATM and TNF-α, we synthesized ATM and TNF knockdown lentiviruses and their controls from Genechem (Shanghai, China). The titers of atm knockdown lentivirus and tnf knockdown lentivirus were 6.5E+8 TU/ml and 9.5E+8, respectively. Firstly, TNBC cells were seeded into 6-well plates the day before transfection (20–30% confluency). The following formula was used to generate lentivirus volume: lentivirus volume (ml) = (MOI × cell count)/viral titers, where MOI indicates multiplicity of infection and cell count is how many cells in the plates. knockdown lentivirus was infected into TNBC cell lines MDA-MB-231 and HCC1937 according to the manufacturer's protocol. 16h after virus infection, cells were treated with Complete-DMEM (CD; DMEM containing 10% FBS and 1% penicillin-streptomycin antibiotic). 72h after virus infection, cells were treated with puromycin (1μg/ml) for 10–14 days and then

collected for analysis. ATM and TNF-α genes knockdown efficiency was determined by using Western blot and qRT-PCR.

RNA oligonucleotides of STAT1 were purchased from Sangon Biotech. And RNA oligonucleotides transfection was performed using Lipofectamine 3000 reagent (Invitrogen). 48h after RNA oligonucleotides transfection, cells were used for molecular assays. The target sequences were listed in Table S1.

Western blot

Cells were lysed in RIPA buffer (MedChemExpress) containing 1.0mM PMSF. After boiling at 95°C for 10min, the proteins were separated on 10% SDS-PAGE gel and then transferred to PVDF membranes (cytivaAmersham). After the blocking procedure, membranes were incubated with primary antibodies (1:1000), visualized in Imager (Bio-Rad) using the ECL system (Thermo Fisher Scientific).

Primary antibodies were: anti-ATM (Abcam, ab32420), anti-ATM (phosphoS1981) (Abcam, ab81292), anti-TNF-α (Abcam, ab1793), anti-MHC class I + HLA A + HLA B (Abcam, ab134189), anti-STAT1(Cell Signaling Technology, #14994), anti-Phospho-Stat1 (Tyr701) (Cell Signaling Technology, #7649), anti-β-actin (Cell Signaling Technology, #4970), anti-p-c-Jun (Santa Cruz Biotechnology, sc-822), anti-c-Jun(Santa Cruz Biotechnology, sc-74543). Secondary antibodies were: anti-rabbit IgG, HRP-linked antibody (Cell Signaling Technology, #7074),

Anti-mouse IgG, HRP-linked Antibody (Cell Signaling Technology, #7076).

RNA extraction and quantitative reverse transcription-PCR (qRT-PCR)

Total RNA was isolated from cells using Eastep® Super Total RNA Extraction Kit RNA (Promega, LS1040). Total RNA (1μg) was used to synthesize cDNA using PrimeScript™ RT reagent Kit with gDNA Eraser (Perfect Real Time) (Takara, RR047A). qRT-PCR was conducted using the appropriate primers and a Bio-Rad CFX96 system with SYBR green to determine the mRNA levels of genes of interest using the following protocol: 95°C for 30s, followed by 40 cycles of 95°C for 5s, 55°C for 30s, and 72°C for 30s. Each sample was detected in triplicate. Expression levels were normalized to β-actin mRNA levels. The primer sequences for ATM, TNF-α, HLA-A, B2M, TAPBP, ERAP1, TAP1, TAP2, NLRC5 and GAPDH were listed in Table S2. Melting curve analysis was performed to monitor PCR product purity and the $2^{-\Delta\Delta Ct}$ method was used to quantify the expression of these indicated genes.

Animal experiments

All animals were cared for in compliance with the principles and procedures of the NIH Guide for the Care and Use of Laboratory Animals, and animal experiment procedures were approved by the Ethics Committee of Tianjin Medical University Cancer Institute and Hospital. 4-6-week-old female BALB/c mice were obtained from the SPF BIOTECHNOLOGY CO.LTD (BEIJING). Wild type 4T1 cancer cells (1 ×

10^6) were injected to the fat pads of female mice. Tumor volume was measured at the indicated time points starting at one week post tumor injection. The following formula was used to generate volume: Volume (mm^3) = 0.5 × length × width^2, where length indicates the longest dimension and width is perpendicular to length. When the tumor volume reached about 100 mm^3, mice received a 10 mg/kg dose of KU55933 or placebo via intraperitoneal injection every three days. Mice received intraperitoneal injections of 100μg of anti-PD-1 (clone 29F.1A12™; Bio X Cell) per injection or its isotype control (clone 2A3; Bio X Cell) on days 10, 17 and 24. Radiotherapy was administered when the tumor volume of the mice reached approximately 300 mm^3. RT in vivo was conducted using the RS2000 Biological X-ray irradiator. Local tumor RT was delivered to the exposed tumor by shielding the rest of the mouse using custom lead jigs. Until the tumor volume reached the size where it should be sacrificed, the mice were euthanised to remove the tumor. The tumor tissues were formalin-fixed, paraffin-embedded, and sectioned for further analysis.

Evaluation of immunohistochemistry

The staining intensity of ATM, p-ATM^{S1981}, TNF-α, p-STAT1 and MHC-I (known as HLA in humans and H-2 in mice) was semi-quantitatively divided into negative, weak (1), moderate (2) and strong (3), An H-score (Staining intensity × Percentage of positive cells) was calculated after recording the percentage of stained cells [1]. In humans with immunohistochemical H-score above the median (ATM, H-score ≥ 60; TNF-α, H-score ≥ 60; p-STAT1, H-score ≥ 45; For HLA, H-score ≥ 70) were judged

as high levels of expression for the relevant molecules, while cases with H-scores below the median were regarded as low levels of expression. In mice with immunohistochemical H-score above the median (p-Atm, H-score ≥ 80; Tnf-α, H-score ≥ 90; p-Stat1, H-score ≥ 85; For H-2, H-score ≥ 80) was defined as high expression of relevant molecules, while cases with H-scores below the median were regarded as low levels of expression.

According to the 2014 International TILs (Tumor-Infiltrating Lymphocytes) Working Group criteria, TILs were graded and divided into two groups based on the median (10%) [2]. CD8 was stained on the cell membranes and nuclei of tumor stromal infiltrating lymphocytes respectively. Ten fields were randomly selected under high magnification (400 ×). The number of positive cells in each field was calculated, and then the average number of positive cells in the 10 fields was calculated.

Statistical analysis

Statistical analysis was performed using Microsoft Office Excel, GraphPad Prism8 version and SPSS 26.0 software. Experiments were performed in triplicate and repeated at least twice. Bars indicate the mean ± standard deviation (SD) of three independent replicates. Differences in mean values between the two groups were analyzed by two-tailed Student's t-test. The Spearman rank correlation test was used for the association analyses between two skew-distributed markers. Tumor growth curves were analysed by repeated measure two-way ANOVA (time × tumor volume).

Survival curves were analyzed by log-rank test. Each experiment was conducted independently at least three times. $P < 0.05$ was considered statistically significant.

REFERENCES

1. Sun M, Guo X, Qian X, Wang H, Yang C, Brinkman KL, et al. Activation of the ATM-Snail pathway promotes breast cancer metastasis. J Mol Cell Biol. 2012;4:304-15.
2. Salgado R, Denkert C, Demaria S, Sirtaine N, Klauschen F, Pruneri G, et al. The evaluation of tumor-infiltrating lymphocytes (TILs) in breast cancer: recommendations by an International TILs Working Group 2014. Ann Oncol. 2015;26:259-71.
