# Supplementary material for: Inhibition of ATM enhances the immunogenicity of triple-negative breast cancer by promoting MHC-I expression
Source: Cell Death Dis. 2025 Aug 18;16(1):624. doi: 10.1038/s41419-025-07944-y (PMC12361503; doi:10.1038/s41419-025-07944-y)
Supplement: Supplementary file 2 — western blot raw data [file 41419_2025_7944_MOESM2_ESM.pptx]

## Slide 1
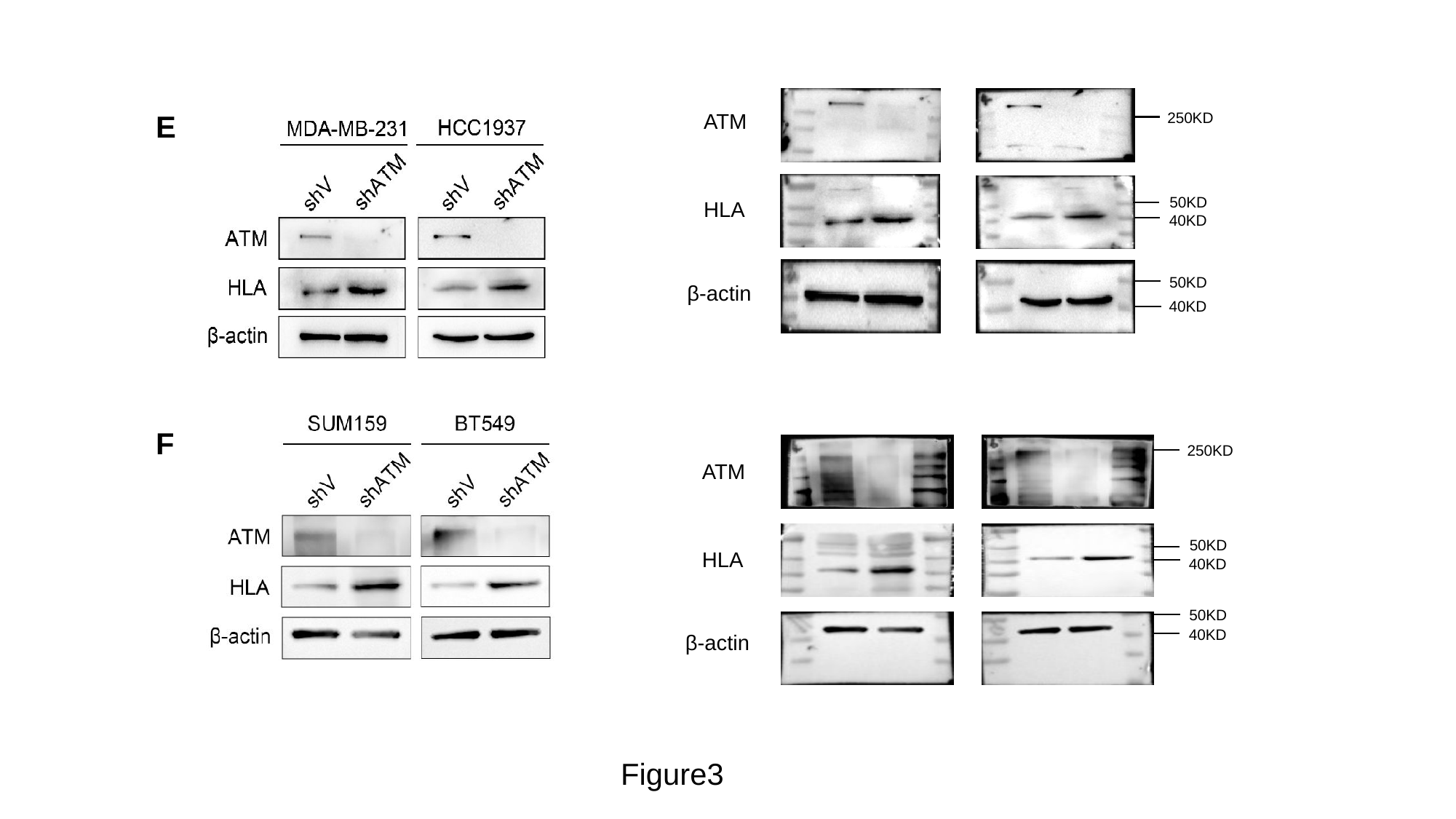

E
250KD
ATM
50KD
HLA
40KD
50KD
β-actin
40KD
F
250KD
ATM
50KD
HLA
40KD
50KD
40KD
β-actin
Figure3

## Slide 2
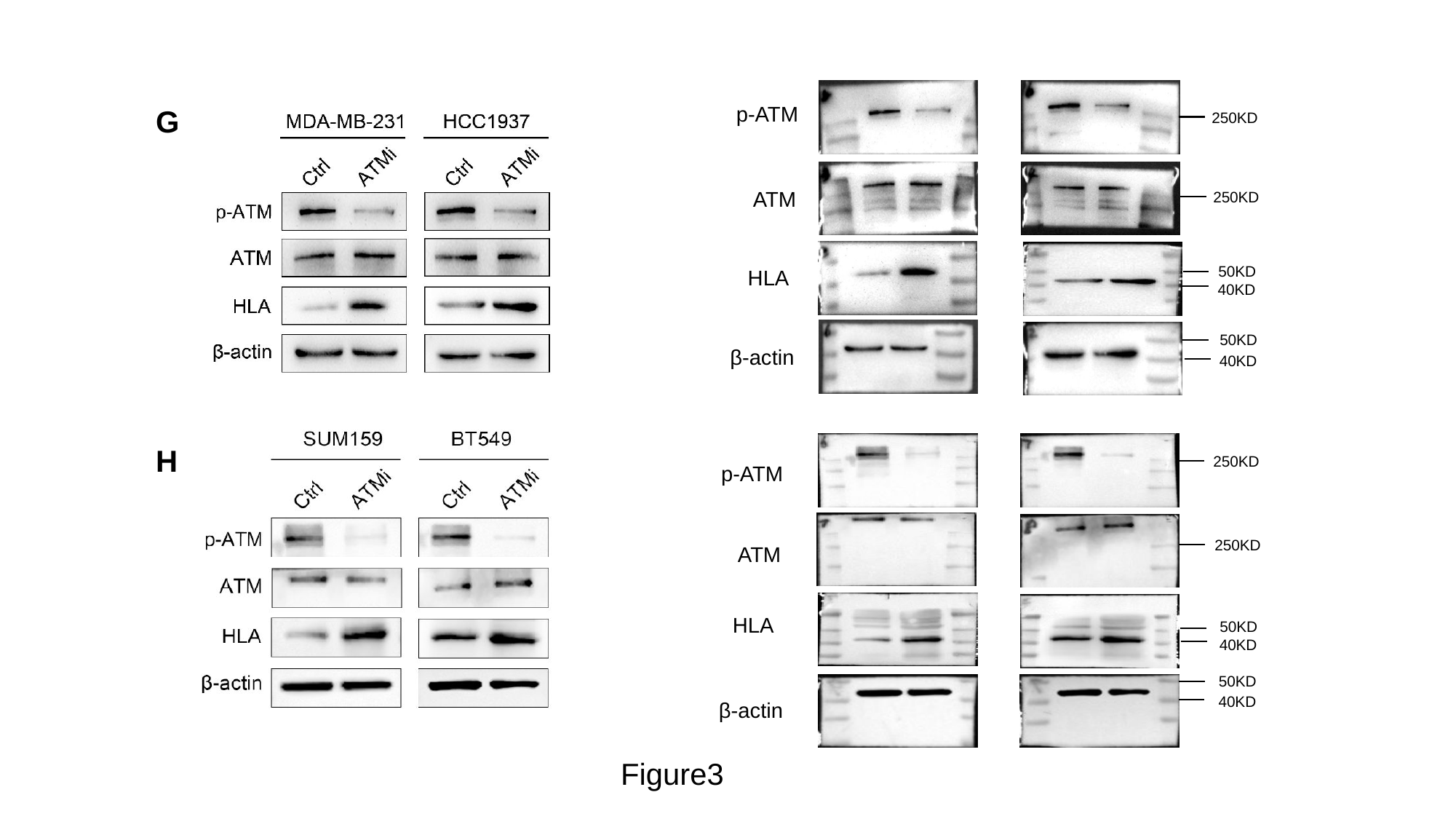

p-ATM
G
250KD
ATM
250KD
50KD
HLA
40KD
50KD
β-actin
40KD
H
250KD
p-ATM
250KD
ATM
HLA
50KD
40KD
50KD
40KD
β-actin
Figure3

## Slide 3
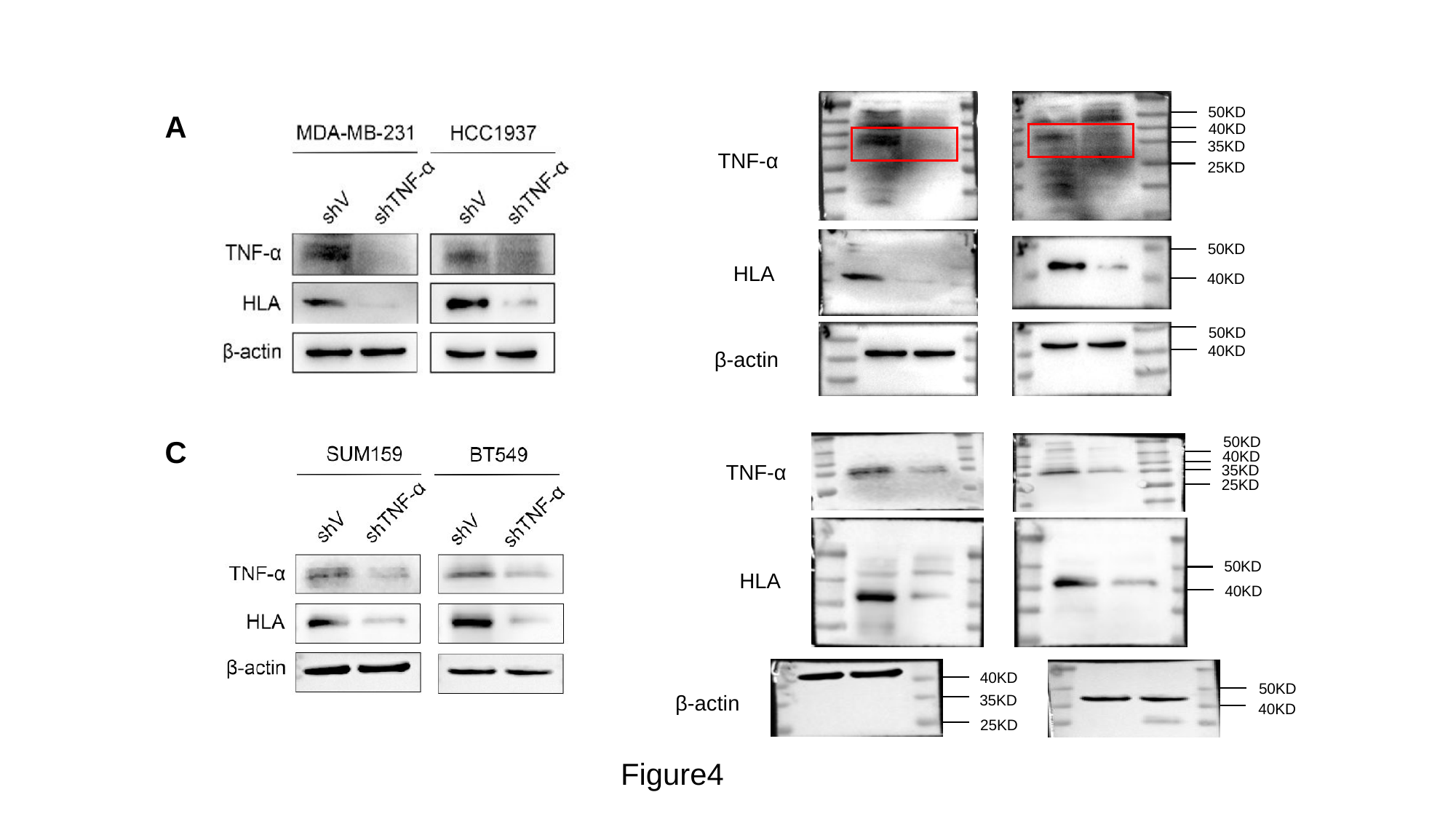

50KD
A
40KD
35KD
TNF-α
25KD
50KD
HLA
40KD
50KD
40KD
β-actin
50KD
C
40KD
TNF-α
35KD
25KD
50KD
HLA
40KD
40KD
50KD
β-actin
35KD
40KD
25KD
Figure4

## Slide 4
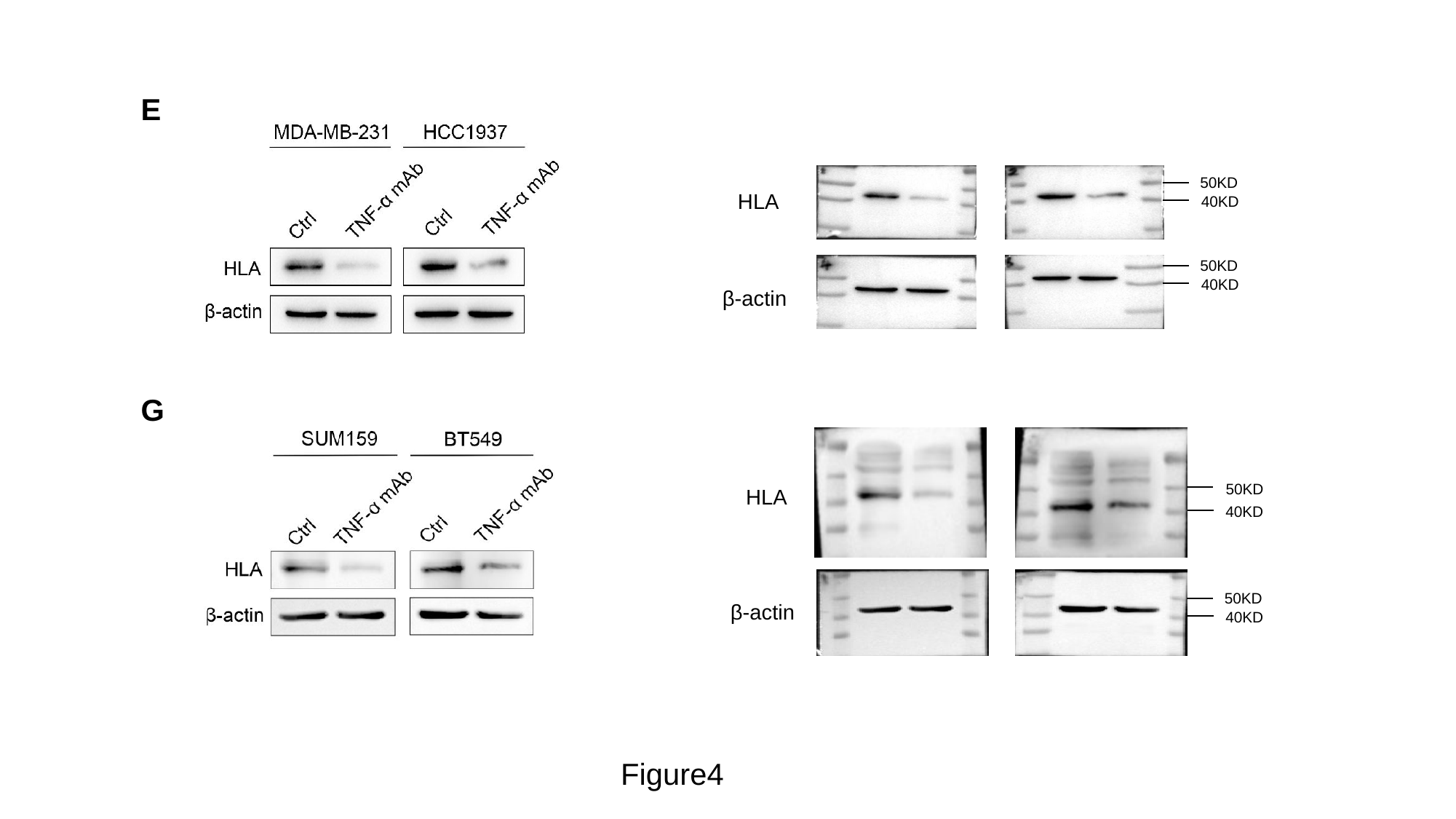

E
50KD
HLA
40KD
50KD
40KD
β-actin
G
50KD
HLA
40KD
50KD
β-actin
40KD
Figure4

## Slide 5
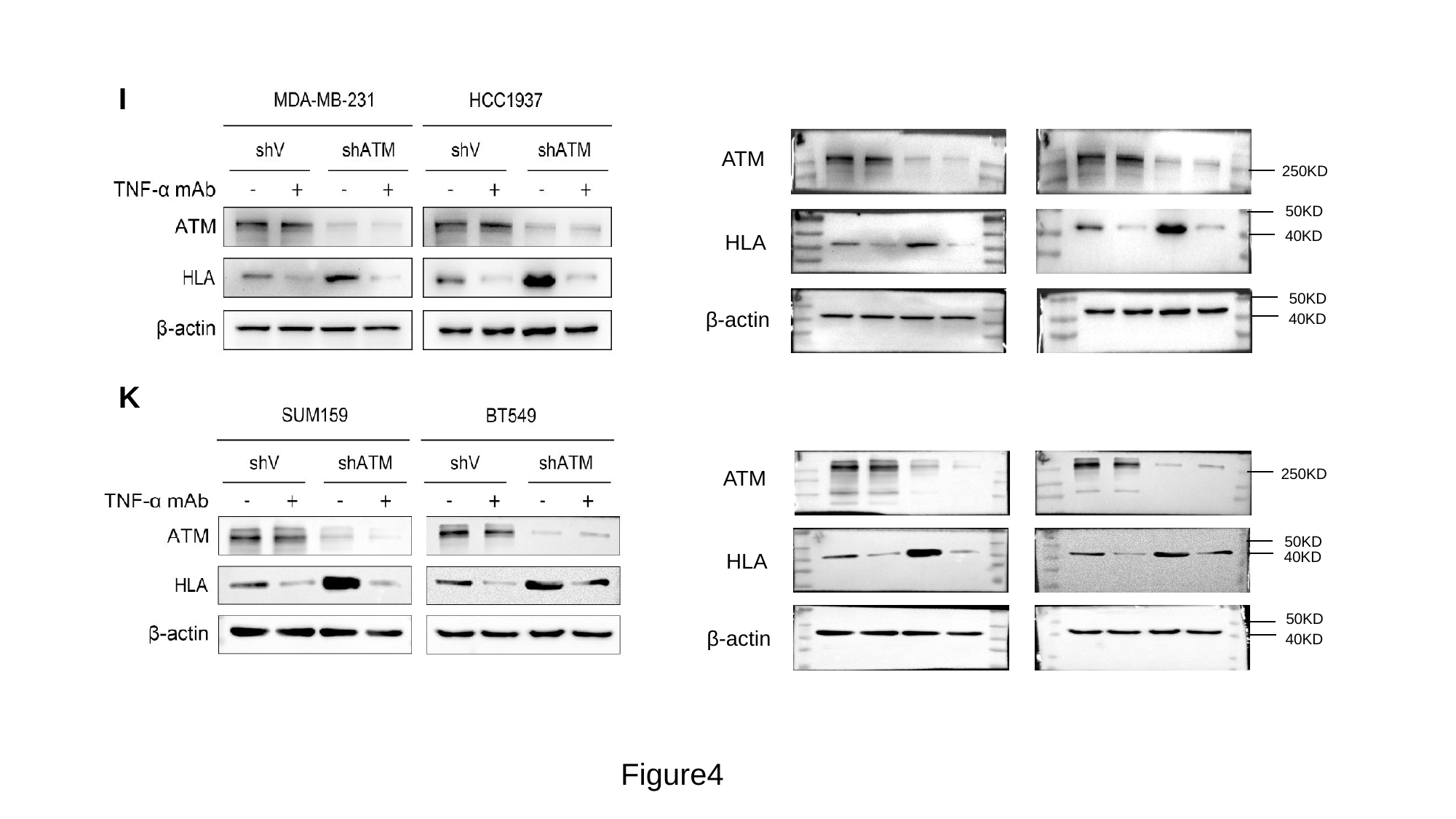

I
ATM
250KD
50KD
40KD
HLA
50KD
β-actin
40KD
K
250KD
ATM
50KD
40KD
HLA
50KD
β-actin
40KD
Figure4

## Slide 6
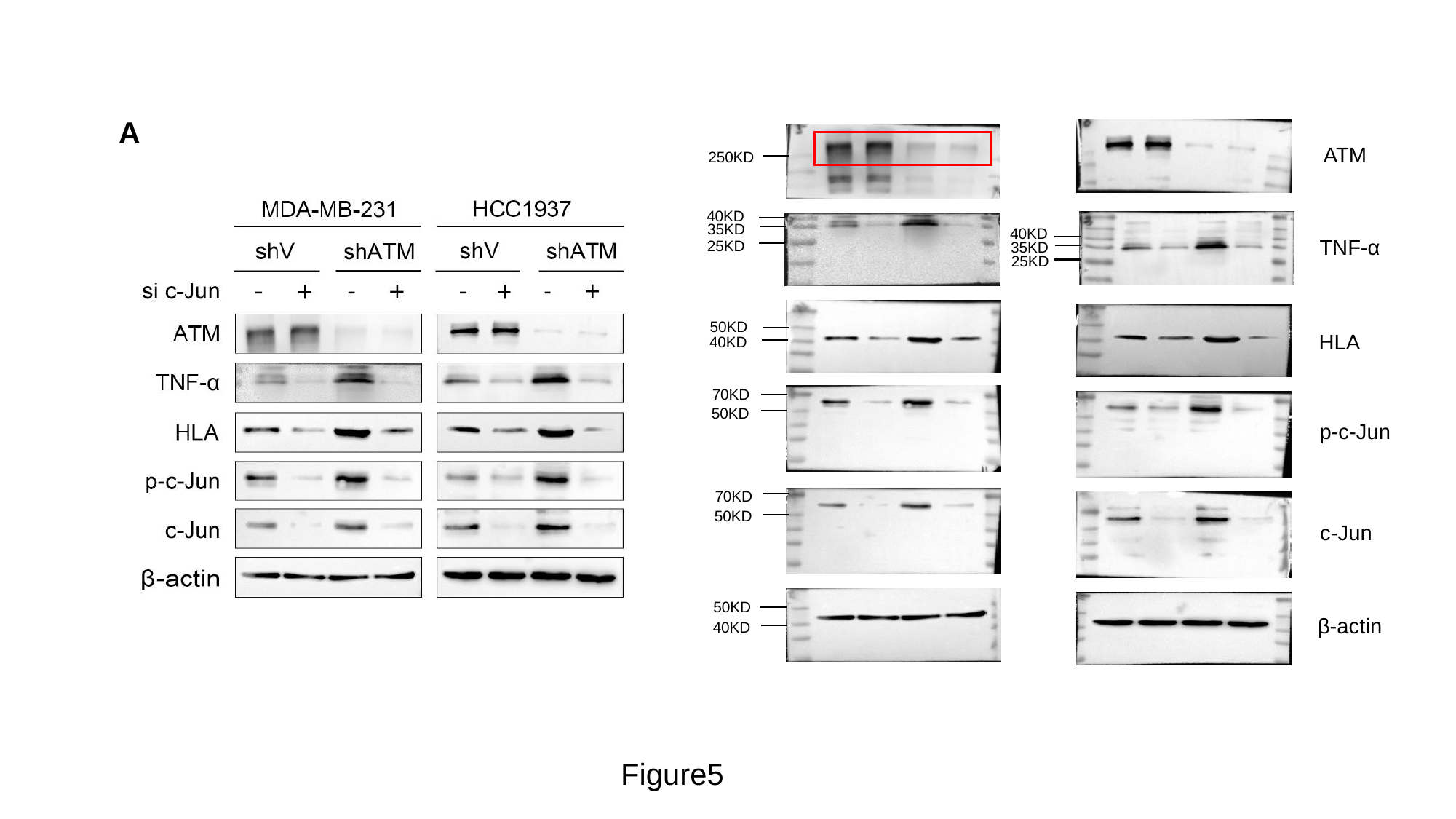

A
ATM
250KD
40KD
35KD
40KD
TNF-α
25KD
35KD
25KD
50KD
HLA
40KD
70KD
50KD
p-c-Jun
70KD
50KD
c-Jun
50KD
β-actin
40KD
Figure5

## Slide 7
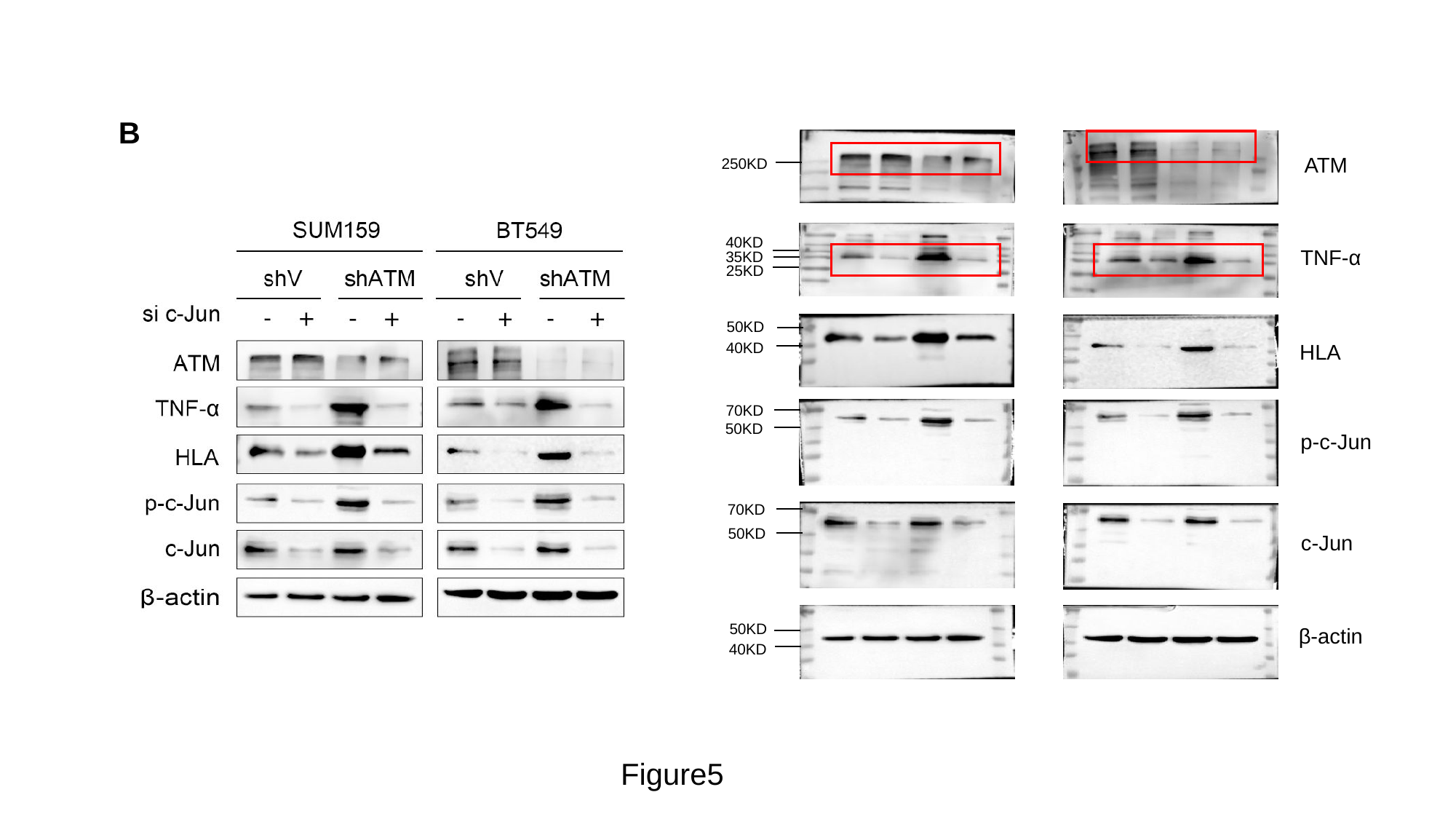

B
ATM
250KD
40KD
TNF-α
35KD
25KD
50KD
40KD
HLA
70KD
50KD
p-c-Jun
70KD
50KD
c-Jun
50KD
β-actin
40KD
Figure5

## Slide 8
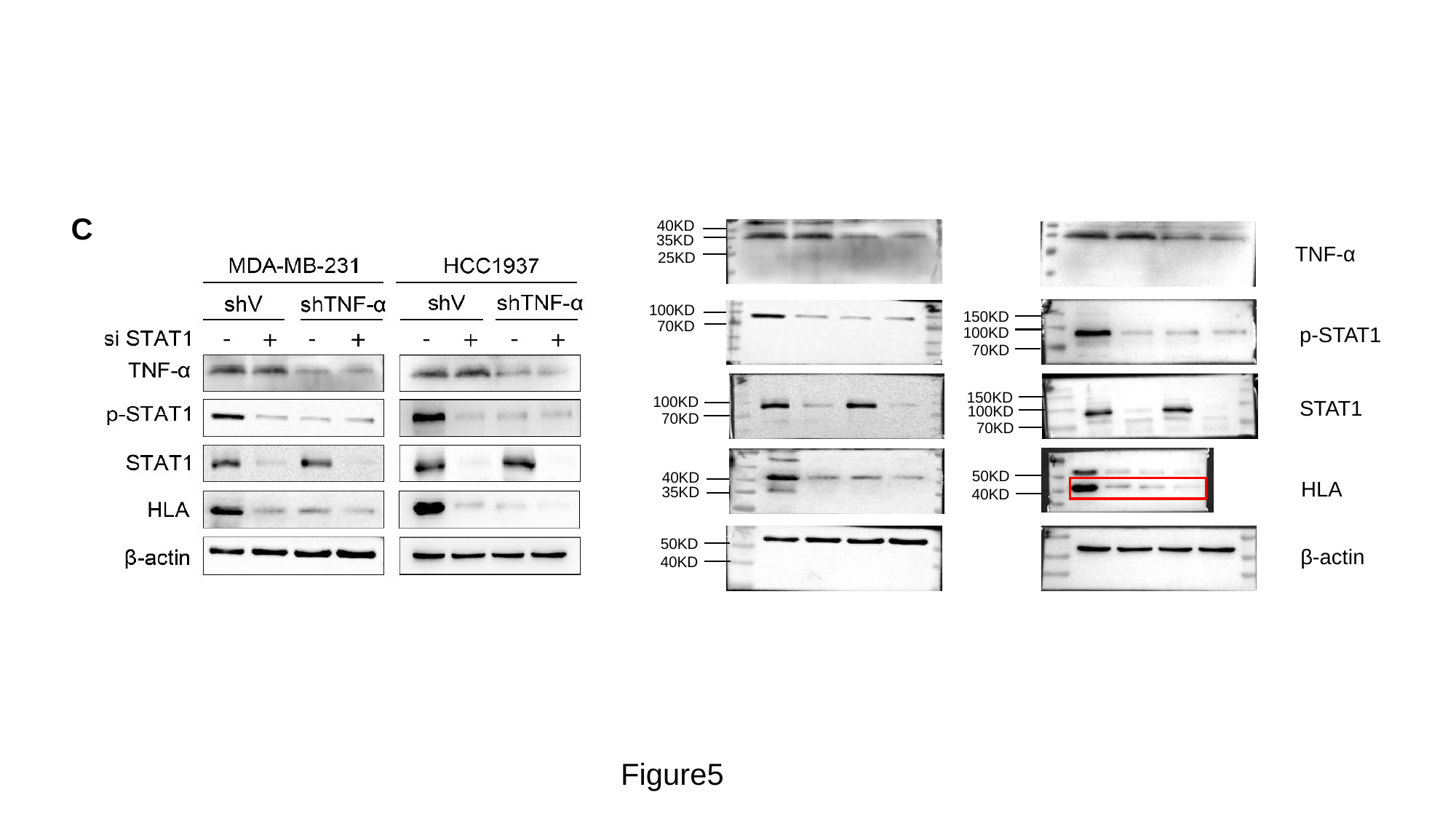

C
40KD
35KD
TNF-α
25KD
100KD
150KD
70KD
p-STAT1
100KD
70KD
150KD
100KD
STAT1
100KD
70KD
70KD
50KD
40KD
HLA
35KD
40KD
50KD
β-actin
40KD
Figure5

## Slide 9
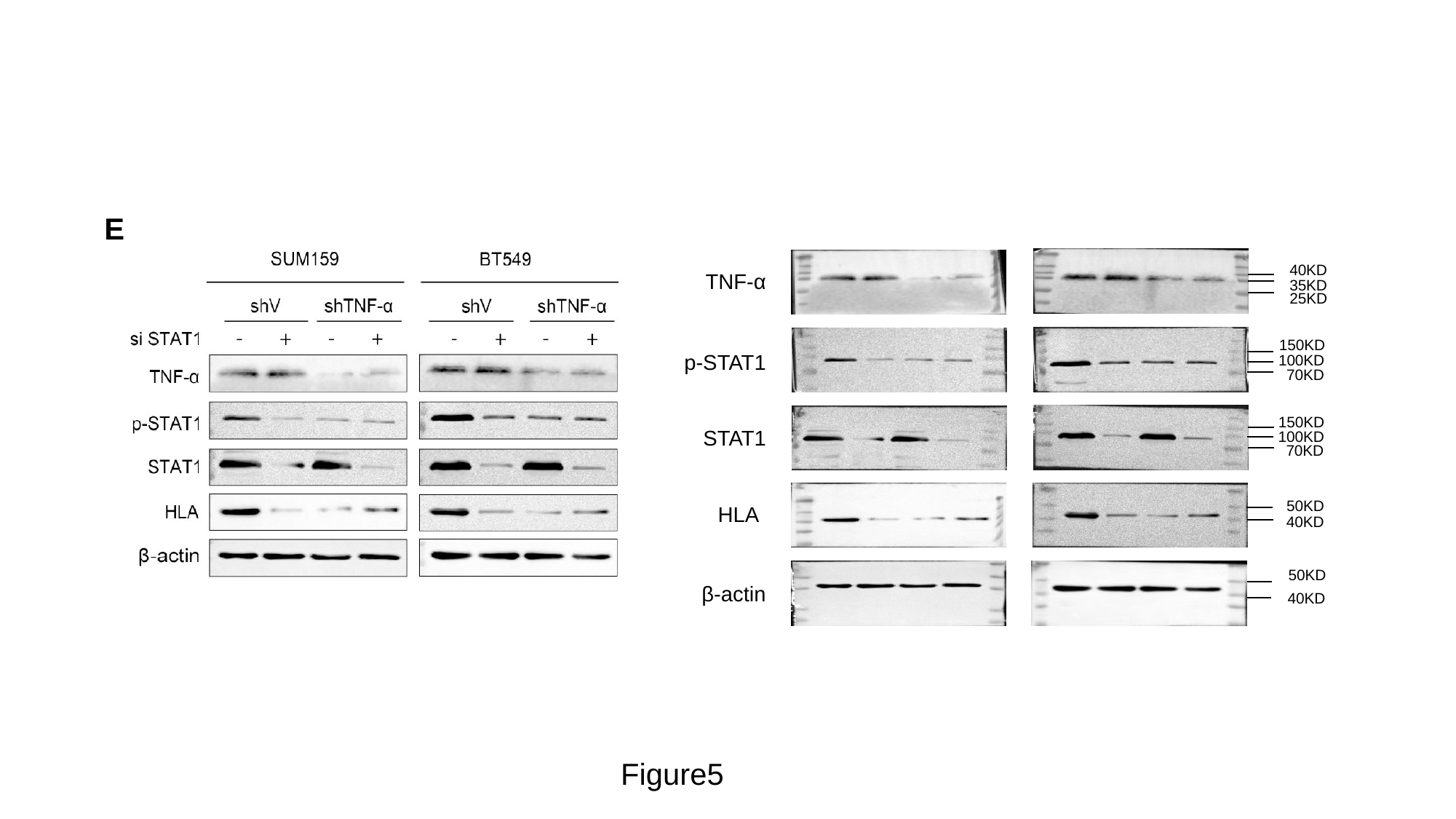

E
40KD
TNF-α
35KD
25KD
150KD
p-STAT1
100KD
70KD
150KD
STAT1
100KD
70KD
50KD
HLA
40KD
50KD
β-actin
40KD
Figure5
